# Down-regulation of the cotton endo-1,4-β-glucanase gene *KOR1* disrupts endosperm cellularization, delays embryo development, and reduces early seedling vigour

**DOI:** 10.1093/jxb/erv111

**Published:** 2015-03-24

**Authors:** Xiaoguang Shang, Qichao Chai, Qinghu Zhang, Jianxiong Jiang, Tianzhen Zhang, Wangzhen Guo, Yong-Ling Ruan

**Affiliations:** ^1^State Key Laboratory of Crop Genetics and Germplasm Enhancement, Cotton Research Institute, Nanjing Agricultural University, Nanjing 210095, China; ^2^School of Environmental and Life Sciences, University of Newcastle, Callaghan, NSW 2308, Australia; ^3^Australia–China Research Centre for Crop Improvement, the University of Newcastle, Callaghan, NSW 2308, Australia; ^4^College of Bioscience and Technology, Hunan Agricultural University, Changsha 410128, China

**Keywords:** Cell wall formation, cotton fibre, endo-1, 4-β-glucanase, endosperm, seed development, seedling vigour.

## Abstract

Towards the aim of examining the potential function of KORRIGAN (KOR), a highly conserved membrane-bound endoglucanase, in reproductive development, here transgenic evidence is provided that a cotton (*Gossypium hirsutum*) endoglucanase, GhKOR1, plays significant roles in endosperm and embryo development. RNA interference (RNAi)- and co-suppression-mediated down-regulation of *GhKOR1* resulted in smaller filial tissue and reduced seed weight, which were characterized by disrupted endosperm cellularization and delayed embryo development, leading to a delayed germination and a weak growth of seedlings early in development. The transgenic seeds exhibited fewer and smaller endosperm cells with irregular and brittle cell walls, and their embryos developed only to the globular stage at 10 days post-anthesis (DPA) when the wild-type endosperm has become highly cellularized and the embryo has progressed to the heart stage. The transgenic seed also displayed a significant reduction of callose in the seed coat transfer cells and reduced cellulose content both in the seed coat and in mature fibres. These findings demonstrate that *GhKOR1* is required for the developmental of both seed filial and maternal tissues and the establishment of seedling vigour.

## Highlight

Transgenic evidence is provided that the expression of a cotton endo-1,4-β-glucanase, GhKOR1, is required for endosperm cellularization, cotton fibre cellulose synthesis, and the establishment of seedling vigour

## Introduction

In addition to the cellulose synthase complex, KORRIGAN (KOR), a plasma membrane-bound endo-1,4-β-glucanase, is another protein required for complete and correct cellulose biosynthesis and cell wall formation (Nicol *et al.*, 1998; Zuo *et al.*, 2000). It has been proposed that KOR may cleave the sitosterol-glucoside (SG) primer from the glucan chain for recycling in β-1,4-glucan polymer synthesis (Peng *et al.*, 2002). An alternative possibility is that KOR1 may edit the growing microfibrils to ensure proper packing of individual chains or to terminate the elongation of the cellulose chains (Taylor, 2008).

KOR was initially isolated from the *Arabidopsis thaliana* dwarf mutant, *kor1-1*, which displayed pronounced architectural alterations in the primary cell wall (Nicol *et al.*, 1998). Mutations in KOR resulted in the reduction of cellulose content in both the primary and secondary cell wall and, hence, various phenotypes in *Arabidopsis* mutants, including dwarfism (Nicol *et al.*, 1998), radial swelling of root tips (Lane *et al.*, 2001), and collapse of xylem vessels (Szyjanowicz *et al.*, 2004). The *altered cell wall 1* (*acw1*) mutant characterized by a mutation in AtKOR1, for example, exhibited impaired tissue elongation in the hypocotyls, roots, and petioles at the non-permissive temperature, indicating a role for KOR1 in cell elongation (Sato *et al.*, 2001). Consistently, mutational studies on OsGLU3, the homologue of AtKOR1 in rice (*Oryza sativa* L.), revealed similar functions of KOR1 in root cell elongation (Zhang *et al.*, 2012). Indeed, the function of KORs appears to be conserved between angiosperm and gymnosperm species (Maloney *et al.*, 2012).

In addition to the function of KOR in cell expansion, it may also play a role in cell division. For instance, the most severe phenotype in the *Arabiddopsis kor1-2* mutant is defective cytokinesis in the shoot apical meristem (SAM), a result of incomplete cell wall formation in the SAM (Zuo *et al.*, 2000). This finding indicates a requirement for KOR for cell division, a view further supported by the analyses on the *tumorous shoot development* (*tsd1*) mutant of *Arabidopsis*. This mutant develops disorganized tumorous-like shoot tissue instead of organized leaves and stems due to a point mutation in the *KOR1* gene (Frank *et al.*, 2002; Krupkova and Schmulling, 2009).

Despite the progress in our understanding of the functions of KOR in vegetative development as outlined above, its potential roles in reproductive organs, such as developing seeds, remains largely unknown. To this end, mutations in cellulose synthase, such as *AtCesA1*, have been shown to cause embryo lethality owing to severe cellulose deficiency in the homozygous mutant embryos (Beeckman *et al.*, 2002; Gillmor *et al.*, 2002). In cotton, suppression of sucrose synthase (Sus), a major enzyme responsible for producing UDP-glucose, the substrate for cellulose biosynthesis, arrested seed development (Ruan *et al.*, 2003). Given the crucial role of cellulose biosynthesis in seed development and the requirement for KOR for this process (Nicol *et al.*, 1998; Zuo *et al.*, 2000), it was hypothesized that KOR may have a major role in seed development. Elucidation of whether and how KOR functions in seed development will not only advance our understanding of the significance of KOR in plant development but could also lead to new ways to improve seed yield and quality, a topic of major significance for global food security (Ruan, 2014).

In this study, the aim was to explore the functions of KOR in developing cotton seed, using a reverse genetic approach. Cotton seed is unique in that its seed coat epidermis produces single-celled fibres for cellulose production whereas its embryo synthesizes oils and proteins (Ruan *et al.*, 2005). As such, cotton seed is perhaps the only seed type where both the maternal and filial tissues are of significant importance to humans and is an ideal system for studying the interaction between maternal and filial tissues (Ruan *et al.*, 2005). First, an orthologue of *AtKOR1* from cotton, *GhKOR1*, was cloned. Following confirmation that *GhKOR1* was strongly expressed in developing cotton seed, homozygous transgenic cotton plants in which *GhKOR1* was suppressed were generated by using the RNA interference (RNAi) or co-suppression approach. Analyses of the transgenic plants revealed several novel observations on the functions of the *KOR1* gene in seed development. Most strikingly, down-regulation of *GhKOR1* disrupted endosperm cellularization and delayed embryo development. These effects led to a smaller filial tissue and reduced mature seed weight, delayed germination, and weakened seedling vigour. Significant reduction of callose in the seed coat transfer cells and reduced cellulose content in mature fibres were also observed. Together, the data provide evidence for the essential role of KOR in the development of seed filial and maternal tissues.

## Materials and methods

### Plant materials and growth conditions

Cotton (*Gossypium hirsutum* cv. Coker 312) plants were grown in a glasshouse with a temperature of 28 °C/20 °C (day/night) and a photoperiod of 14h of light and 10h of dark. Cotton fruit age was determined by tagging each pedicel on the day of flowering. For DNA and RNA extraction and crystalline cellulose content determination, samples were frozen in liquid N_2_ and stored at –70 °C until use. Fresh samples were used for fluorescence microscopy and fibre length measurement.

### Isolation of *GhKOR1* cDNA and sequence analysis

A full-length cDNA encoding the putative *GhKOR1* (GenBank accession no. AY 574906) was obtained from a *G. hirstutum* fibre cDNA library. Gene-specific primers (Supplementary Table S1 available at *JXB* online) were designed according to the cotton fibre expressed sequence tag (EST) sequences homologous to *A. thaliana KOR* genes.

The full-length *GhKOR1* cDNA sequence was analysed using DNAssist 2.0 software. The deduced KOR1 amino acid sequences were downloaded from the NCBI for alignment analysis with Clustal X software (version 1.83) (Thompson *et al.*, 1997). The results were exported and edited by gene DOC software (Nicholas and Nicholas, 1997).

### Gene constructs and cotton transformation

To construct the RNAi vector, primers were designed at the 260th and 822th nucleotides after the ATG of the *GhKOR1* sequence (Supplementary Table S1 at *JXB* online). The amplified 562bp sense and antisense fragments were then subcloned into the pCAMBIA1301 vector with inverse orientations under the control of the constitutive *Cauliflower mosaic virus* (CaMV) 35S promoter. An overexpression construct was also made by cloning the full-length *GhKOR1* cDNA downstream of the 35S promoter in the pCAMBIA1301 vector.

The constructs were introduced into cotton using *Agrobacterium tumefaciens*-mediated transformation as described previously (Li *et al.*, 2009). The homozygosity of transgenic plants was determined by segregation analyses of the kanamycin selection marker coupled with PCR-based genotyping for the presence or absence of the transgene (*35S-GhKOR1*). The primers used for vector construction and genotyping analysis are listed in Supplementary Table S1 at *JXB* online.

### Semi-quantitative and quantitiative real-time PCR analysis

The transcript levels of *GhKOR1* and *GhKOR2* were analysed by both semi-quantitative real-time PCR (RT-PCR) and quantitative RT-PCR (qRT-PCR). The cotton *Gh18srRNA* gene and histone 3 gene *GhHis3* were used as the internal control in semi-quantitative RT-PCR and qRT-PCR, respectively. Total RNA was isolated from cotton fibre and other tissues using the cetyltrimethyl ammonium bromide (CTAB)-acid phenol extraction method followed by reverse transcription into cDNA (Jiang and Zhang, 2003). qRT-PCR was performed using a LightCycler FastStart DNA Master SYBR Green I kit (Roche, Basel, Switzerland) in an CFX96 Touch™ Real-Time PCR detection system according to the manufacturer’s protocol (Bio-Rad, http://www.bio-rad.com). Three biological replicates were used for each reaction with three technical replicates each. The gene-specific primers and conditions used are described in Supplementary Table S1 at *JXB* online. In wild-type (WT) cotton, the relative transcript level of the target gene was calculated by the equation *Y*=2^–ΔCt (where ΔCt is the difference between the Ct values of the target gene products and the *GhHis3* products; that is, ΔCt=Ct_*GhKOR1*_–Ct_*GhHis3*_). When the transcript level of a target gene in the transgenic cotton tissues was compared with that in WT tissues, the equation *Y*=2^–ΔΔCt was applied.

### Seed fixation, embedding, and sectioning

Seed fixation, embedding, and sectioning were conducted according to Pugh *et al.* (2010). In brief, fixed seed samples were dehydrated in a 10% step-graded ethanol series from 30% to 100% at 2h intervals. Samples were then stored in 100% ethanol for 3 d to dehydrate the seeds completely, with ethanol changes every day. Thereafter, they were infiltrated with LR White Resin (ProSci Tech, Inc., Qld, Australia) at room temperature through a 20% step-graded series up to 100% with a 2h interval. The samples were then rotated at room temperature with resin changes every 3 d six times. Infiltrated samples were transferred to fresh resin in gelatine capsules and embedded by polymerization for 24h at 60 °C.

Thin sections (2 μm thick) were cut using a Reichert Ultracut microtome with glass knives. The sections were mounted onto gelatine-coated glass slides and then they were ready for staining.

### Fluorescent labelling for callose and cellulose

To visualize the cellular structure of embedded materials, Toluidine Blue staining was performed according to O’Brien *et al.* (1964). The staining solution was made up of 0.5% (w/v) in 1% Borax buffer (pH 9.0) with 0.2% (w/v) Azure II, and stored in the dark at room temperature. After staining for 30 s, the slides were washed briefly with water and viewed under bright-field using a Zeiss Axiophot microscope (Axio Scope A1, Carl Zeiss Microscopy Ltd, Jena, Germany).

Aniline Blue (Currier and Strugger, 1956) and Calcofluor White (CFW) (Herth and Schnepf, 1980) were employed to label callose and cellulose, respectively. Aniline Blue was prepared in water at 0.05% (w/v) and stored in the dark at 4 °C. CFW was prepared in phosphate-buffered saline (PBS) buffer (pH 7.0) at 0.1% (w/v) and stored in the dark. The slides were incubated for 1h in Aniline Blue or 10min in CFW in the dark followed by a 30 s wash with water or PBS buffer, respectively. The fluorescent signal of Aniline Blue and CFW was viewed immediately under UV light with a Zeiss Axiophot microscope as previously described (Ruan *et al.*, 2008).

### Determination of crystalline cellulose content

The crystalline cellulose content of cotton seed coat and fibre was determined using the method of Upegraff (1969). In brief, 50–100mg of dried samples were digested in acetic nitric reagent for 30min in a boiling water bath to remove most of the non-cellulosic components, followed by three washes with sterilized water. The washed samples were then subject to an overnight hydrolysis with 67% H_2_SO_4_ at room temperature to release glucose. Anthrone (0.2%) dissolved in concentrated sulphuric acid was added into the glucose solution and the samples were placed in a boiling water bath for 16min to complete the reaction. The absorbance (620nm) of the processed samples was proportional to the cellulose content of the original sample. Microcrystalline cellulose (S5504, Energy Chemical, China) was used as a standard for the calibration. Data were collected from three technical replicates in each of at least three biological replicates.

### Determination of seed biomass and fibre length

Cotton bolls were harvested at 20 days post-anthesis (DPA). Fibre, seed coat, and filial tissue were separated on ice for the measurement of their fresh and dry weights. For the latter, fresh samples were dried for 48h at 80 °C to constant weight. About 10 seeds were used for measurement from at least three biological replicates (plants) for each WT and transgenic line. The 100 seed weight and fibre weight per seed were measured at the maturation stage from at least nine bolls derived from three biological samples (plants) for each line.

Fibre length was measured from seeds at 20 DPA as described previously (Gipson and Ray, 1969). In brief, a clump of seeds with fibre attached was placed in boiling water with 2.5% HCl for 3min. The seeds were then transferred onto a glass plate. The fibres were straightened under running water for measurement of their length. For consistency, the measurement was taken at the chalazal end of the seed in all cases. At least 14 samples derived from three bolls were used for each measurement.

## Results

### Cloning of *GhKOR1* and its expression patterns

By using PCR screening, a putative full-length endo-1,4-β-glucanase cDNA sequence of 2293bp was isolated from a λZAP11 cDNA library prepared from RNA isolated from developing cotton seed and fibre. The encoded protein sequence, comprised of 619 amino acids, is highly homologous to KORRIGAN1 in *A. thaliana* and hence was designated as GhKOR1.

The *GhKOR1* gene shares a similar structure with *AtKOR1*, both of which contain the same number of exons and introns with similar nucleotide number in the exons (Supplementary Fig. S1 at *JXB* online). Protein alignment analysis showed high identities between GhKOR1 and seven other KOR orthologues from various species (Supplementary Fig. S2). For example, GhKOR1 shared 89, 87, and 82% identities, respectively, with PtrKOR1 in aspen (*Populus tremuloides*) (Bhandari *et al.*, 2006), LeCel3 (SlCel3) in tomato (*Solanum lycopersicum*, formerly *Lycopersicon esculentum*) (Brummell *et al.*, 1997), and AtKOR1 in *A. thaliana* (Nicol *et al.*, 1998). The deduced GhKOR1 protein contains the conserved polarized signal peptide (LL and YXXФ motifs) targeted to the plasma membrane (Zuo *et al.*, 2000), a predicted transmembrane domain, glycosylation sites, and residues essential for catalytic activity (Supplementary Fig. S2). These are hallmarks of functional KOR proteins (Molhoj *et al.*, 2002). Collectively, the analyses strongly indicate that *GhKOR1* encodes a KOR protein in cotton.

**Fig. 1.**
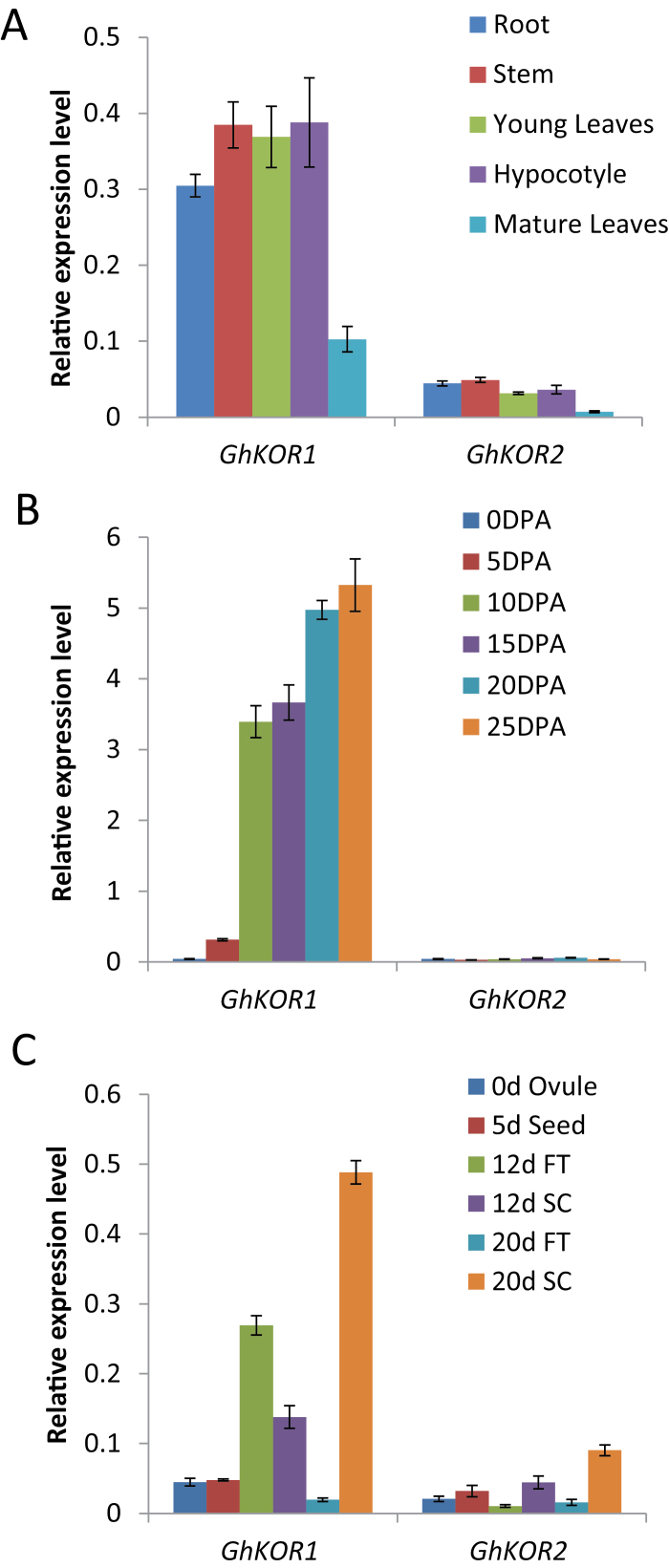
Transcript levels of *GhKOR1* and *GhKOR2* in wild-type cotton measured by qRT-PCR. (A) Transcript levels of *GhKOR1* and *GhKOR2* in root (R), stem (S), young leaves (YL), hypocotyls (H), and mature leaves (ML). (B) Transcript levels of *GhKOR1* and *GhKOR2* in 0, 5, 10, 15, 20 and 25 DPA fibre. (C) Transcript levels of *GhKOR1* and *GhKOR2* in 0 DPA ovule, 5 DPA seed, 10 DPA seed coat (SC) and filial tissue (FT), and 20 DPA seed coat (SC) and filial tissue (FT). (This figure is available in colour at *JXB* online.)

The expression of *GhKOR1* in various tissues was investigated by qRT-PCR. The analyses revealed that *GhKOR1* was highly expressed in actively growing tissues, including developing root and stem, young leaves, and hypocotyl, but was weakly expressed in mature leaves (Fig. 1A). This expression pattern of *GhKOR1* is consistent with that of *AtKOR1* in *Arabidopsis* (Nicol *et al.*, 1998), indicating its role in cell growth. The weak expression of *GhKOR2* (GenBank accession no. HM462003) in these tissues is noteworthy (Fig. 1A); *GhKOR2* is the only remaining gene encoding membrane-anchored endo-1,4-β-glucanase besides *GhKOR1* in the cotton sequence database. Similar results were obtained using semi-quantitative RT-PCR prior to the qRT-PCR analyses (data not shown).

In the developing fibre, the mRNA level of *GhKOR1* was low in early stages but was dramatically increased at ~10 DPA onwards and maintained at a high level up to at least 25 DPA (Fig. 1B), corresponding to a period of rapid elongation (10–20 DPA) and extensive secondary cell wall synthesis from 20 to 25 DPA (Ruan, 2005). Similar to the situation in vegetative tissues, *GhKOR2* was expressed at a much lower level than *GhKOR1* in developing fibres (Fig. 1B).

During cotton seed development, cellulose biosynthesis is required for seed coat transfer cell (TC) wall ingrowths (Wang and Ruan, 2012) and endosperm cellularization (Ruan *et al.*, 2008; Wang and Ruan, 2012). This raises the possibility that GhKOR1 may be involved in these developmental processes. Therefore, *GhKOR1* transcript levels were further examined in dissected seed tissues. Figure 1C shows that the *GhKOR1* gene was highly expressed in 12 DPA filial and seed coat tissues and 20 DPA seed coat when they undergo endosperm cellularization and TC wall ingrowth, respectively (Ruan, 2007; Pugh *et al.*, 2010). This observation (Fig. 1C) implies potential roles for GhKOR1 in these processes. Similar to the case in vegetative and fibre tissues (Fig. 1A, B), the mRNA level of *GhKOR2* was much lower than that of *GhKOR1* in seed coat and filial tissues (Fig. 1C), indicating that GhKOR1 is the major KOR isoform expressed in these tissues.

### Down-regulation of *GhKOR1* inhibits seed growth

The high expression of *GhKOR1* in 12 DPA filial tissue and 20 DPA seed coat (Fig. 1C) led to investigation of the possible roles of *GhKOR1* in seed development. To achieve this goal, *GhKOR1* RNAi and overexpression constructs, both driven by the constitutive 35S promoter, were made. The constructs were introduced into cotton via *Agrobacterium*-mediated transformation using an established protocol (Li *et al.*, 2009). Comprehensive PCR-based genotyping and expression analyses of *GhKOR1* mRNA identified eight RNAi and three co-suppression primary transformants. The screening did not detect any overexpression lines, probably due to sequence homology-dependent co-suppression. Based on the level of suppression of the *GhKOR1* transcript, two RNAi lines and one co-suppression line, designated as RNAi line1, RNAi line5, and Co-S line1, were selected for detailed analyses. These three transgenic lines were self-pollinated to obtain their homozygous progeny. The homozygosity for the transgene was confirmed by PCR at the T_3_ generation onwards. T_5_ homozygous lines were used in the subsequent studies. Since the 562bp *GhKOR1* fragment used in the RNAi construct shared high homology with *GhKOR2* in some regions (Supplementary Fig. S3A at *JXB* online), *GhKOR2* might also be suppressed along with *GhKOR1*. Thus, the transcript level of *GhKOR2* was also measured in the analyses.

**Fig. 2.**
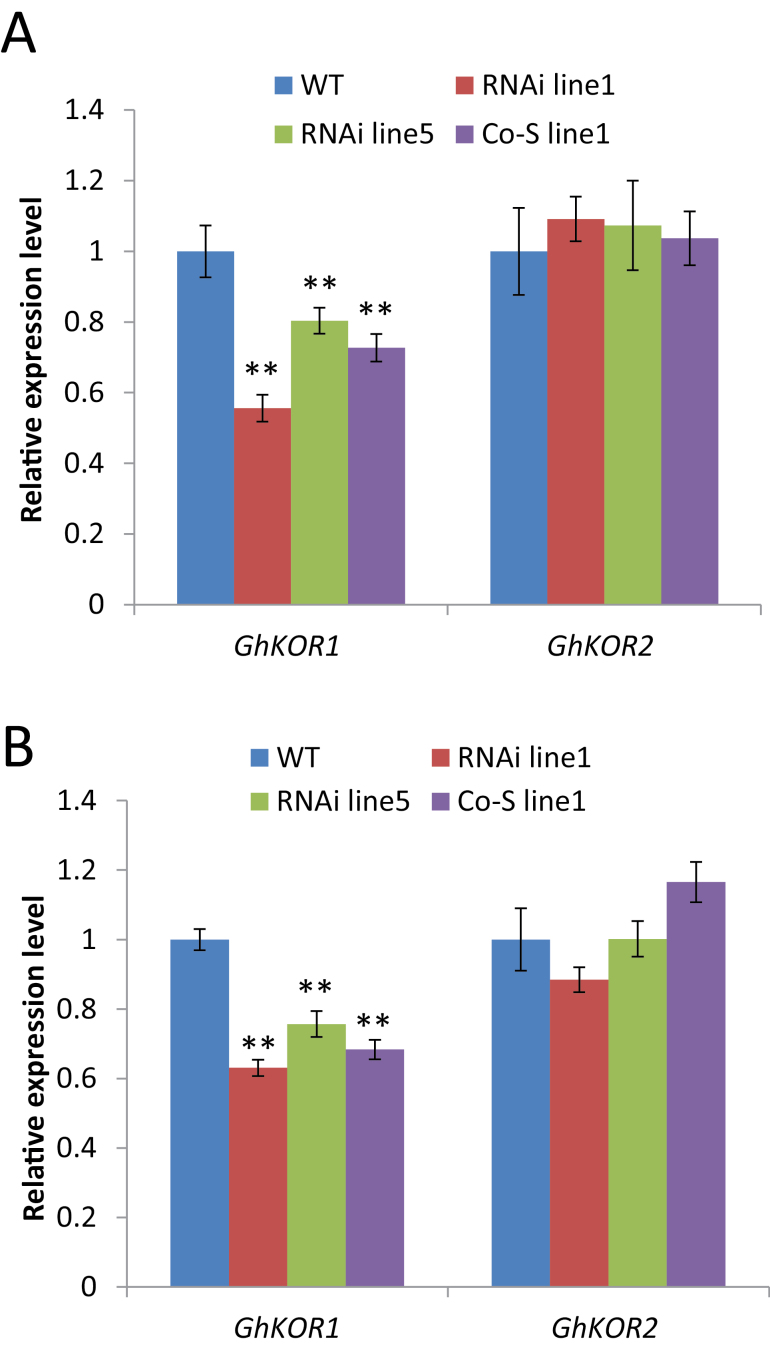
Transcript levels of *GhKOR1* and *GhKOR2* in 12 DPA filial tissue (A) and seed coat (B) of wild-type (WT), RNAi, and co-suppression (Co-S) lines measured by using qRT-PCR. ** indicate a significant difference from the WT based on *t*-test with a *P*-value of 0.01. (This figure is available in colour at *JXB* online.)

From 10 DPA onwards, the cotton seed endosperm is undergoing extensive cellularization (Wang and Ruan, 2012) and TCs begin to initiate wall ingrowth in the seed coat (Pugh *et al.*, 2010). Both of these processes are dependent on active cellulose biosynthesis. Thus, the transcript levels of *GhKOR1* and *GhKOR2* were examined in these tissues. Figure 2A shows that the mRNA level of *GhKOR1*, but not of *GhKOR2*, was evidently down-regulated in 12 DPA filial tissue of all three transgenic lines relative to that of the WT. Similar results were obtained for 12 DPA seed coat, in which the *GhKOR1* gene was reduced by 37, 24, and 32% in RNAi line1, RNAi line5, and Co-S line1, respectively, compared with the WT (Fig. 2B). As in the filial tissues, the mRNA level of *GhKOR2* was only marginally affected in the seed coat, with no significant difference from that of the WT (Fig. 2B). These results indicate that the silencing effect appears to be specific to *GhKOR1*, and any phenotype observed is therefore attributable to reduction of *GhKOR1* expression.

**Fig. 3.**
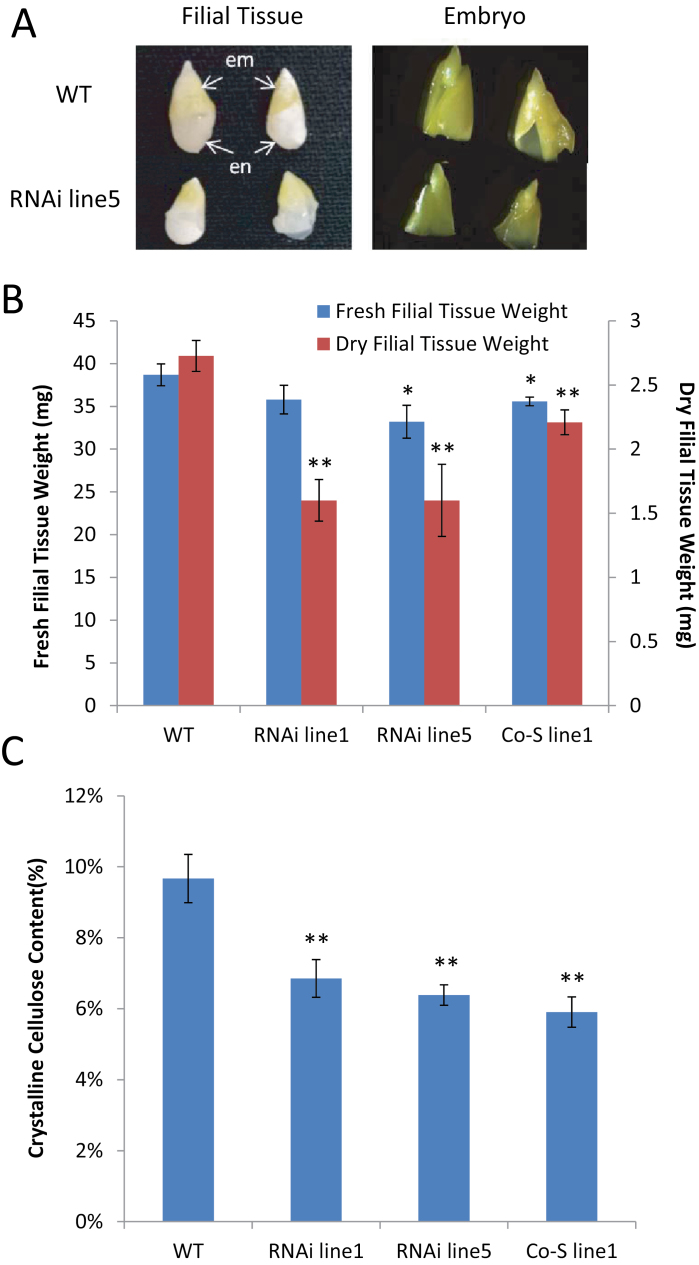
Seed phenotype at 20 DPA in *GhKOR1*-down-regulated transgenic cotton. (A) Filial tissue of RNAi line5 was smaller than that in the WT. A similar phenotype was observed in the other two *GhKOR1*-down-regulated lines, RNAi line1 and Co-S line1. em, embro; en, endosperm. (B) The dry weight and, to a lesser extent, the fresh weight of the 20 DAP filial tissue were reduced in the transgenic cotton, compared with the WT. (C) The crystalline cellulose content of 20 DAP seed coat was reduced in the transgenic cotton compared with the WT. * and ** indicate a significant difference from the WT by *t*-test with *P*-values of 0.05 and 0.01, respectively. At least three biological replicates were used in each case. (This figure is available in colour at *JXB* online.)

By 20 DPA when the seed has fully expanded, it was evident that the filial tissue of the transgenic seed, including its embryo, was much smaller than that of the WT (Fig. 3A). Indeed, the fresh and dry weights of the filial tissue from the transgenic lines were reduced as compared with those in the WT, except for the fresh weight in RNAi line1 (Fig. 3B). Noticeably, the dry weight of filial tissue was reduced much more than their fresh weight, by 41% in RNAi lines1 and 5 and by 19% in Co-S line1. In contrast to the significant reduction in filial tissue dry weight, there was only a 5% reduction in fresh or dry weight of the seed coat in RNAi line1 and no reduction in the other transgenic lines (Supplementary Fig. S4 at *JXB* online). These results indicate that down-regulation of the *GhKOR1* gene impacts more on filial tissue development than on that of the seed coat.

Although the transgenic lines showed little change in the fresh and dry weighs of the 20 DPA seed coat, compared with the WT, the crystalline cellulose content in this tissue was significantly reduced in the transgenic lines, by 29, 34, and 39% in RNAi line1, RNAi line5, and Co-S line1, respectively (Fig. 3C). These data suggest that GhKOR1 also plays a role in cellulose biosynthesis in the 20 DPA seed coat. As seed coat TC wall ingrowths require biosynthesis of cellulose and callose (Pugh *et al.*, 2010; Wang and Ruan, 2012), down-regulation of *GhKOR1* in 20 DPA seed coat may negatively impact on TC development.

### Suppression of GhKOR1 disrupts endosperm cellularization, delays embryo development, and reduces callose deposition in transfer cells

**Fig. 4.**
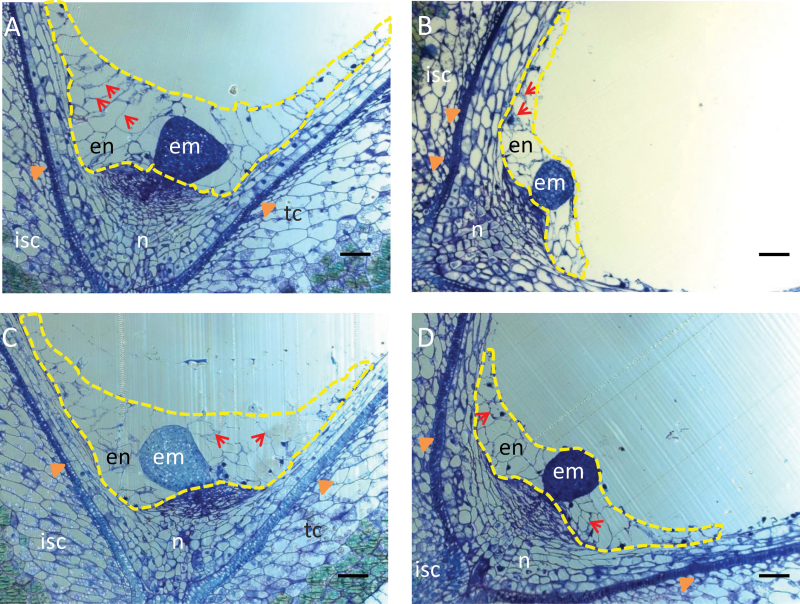
Disrupted endosperm and embryo development in *GhKOR1*-down-regulated transgenic cotton. The resin-embedded longitudinal sections cut from 10 DPA seeds were stained with Toluidine Blue. The endosperms are enclosed by yellow dotted lines. (A) WT seed section. Note that the endosperm was cellularized and well organized with cell walls evidently visible between the cells (arrows), and the embryo has developed to the heart stage. (B) RNAi line1 seed section. Note the much reduced endosperm cell size and cellularization. The latter is indicated by the irregular cell walls as compared with the WT. Also different from that in the WT (A), the embryo is still at the globular stage. (C) RNAi line5 seed section exhibiting similar endosperm and embryo phenotype to those of RNAi line1 in (B). The overall area of the endosperm appeared similar to that in the WT (A). However, the cells are irregular in shape (arrows) and the embryo is at the globular stage. (D) Co-S line1 10 DPA seed section. Note the similar phenotype to that of the RNAi line1 in (B) characterized by smaller and less cellularized endosperm cells and the fact that the embryo is at the globular stage. en, endosperm; em, embryo; n, nucellus; isc, inner seed coat; tc, transfer cell indicated by arrowheads. Three biological replicates were observed for each line, with similar results. Representative images are provided. Scale bars=50 μm in A–D.

To gain insights into the cellular basis underlying the small filial tissue phenotype observed in the transgenic seeds (Fig. 3), histological analyses were conducted on 10 DPA seed sections. The most striking observation was the much reduced and disrupted cellularized endosperm in transgenic seed (Fig. 4B–D) as compared with the WT (Fig. 4A). Similar to seeds of most plant species, cotton seed endosperm development starts with free nuclear divisions without cytokinesis, which is then followed by the cellularization process characterized by cell wall formation (Schulz and Jensen, 1977; Ruan *et al.*, 2008; Wang and Ruan, 2012). The endosperm cellularization starts from the micropylar end and progresses towards the chalazal region (Ruan *et al.*, 2008). For RNAi line1 (Fig. 4B) and Co-S line1 (Fig. 4D), the overall endosperm size was evidently reduced, with a smaller cell size, and their cell number decreased to 65% and 37% of the WT level (the cell number being 85.00±5.51, 55.67±11.57, and 31.67±7.97 per section in the WT, RNAi line1, and Co-S line1, respectively). For RNAi line5, although its endosperm cell number (79.50±5.31 per section) displayed no significant reduction (Fig. 4C), its cell wall structure appeared to be brittle with irregular and distorted shape, compared with the WT (Fig. 4A).

A delayed embryo development was also observed in transgenic seeds. By 10 DPA, the WT embryo has developed to the heart stage (Fig. 4A). However, the transgenic embryos were still at the early globular stage in RNAi line1 and Co-S line1 (Fig. 4B, D) or the later globular stage in RNAi line5 (Fig. 4C).

It is noteworthy that the reduction of the transcript level of *GhKOR1* in the filial tissue of the three transgenic lines matches the degree of inhibition of filial tissue development. For instance, in RNAi line1, the mRNA of *GhKOR1* was reduced most strongly (44% reduction, Fig. 2A) and the filial tissue showed the most severe phenotype among the three transgenic lines (Fig. 4B). On the other hand, RNAi line5 exhibiting weak reduction in the *GhKOR1* mRNA level (Fig. 2A) showed a less obvious filial tissue phenotype as compared with RNAi line1 (Fig. 4C).

Although the *GhKOR1* gene transcript was reduced in 12 DPA seed coat of the transgenic plants (Fig. 2B), no significant morphological difference could be found in this tissue (Fig. 4).

**Fig. 5.**
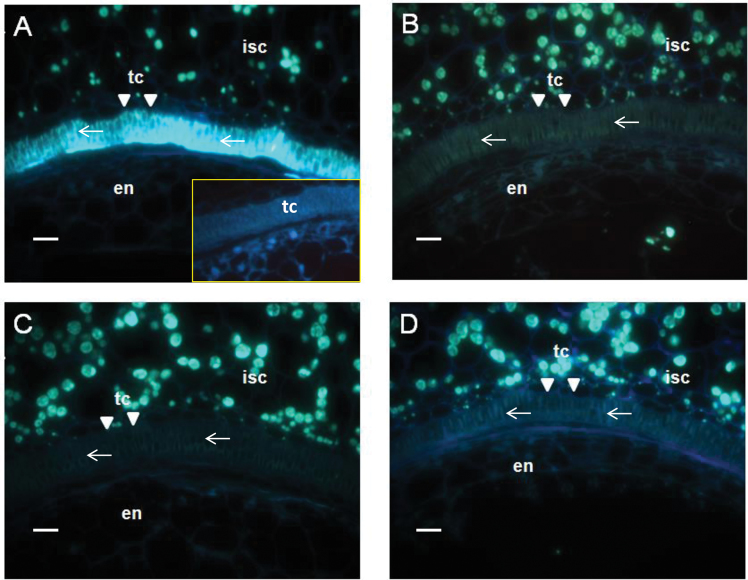
Reduced callose signals in 15 DPA seed transfer cells (TCs) in *GhKOR1*-down-regulated transgenic cotton. Resin-embedded seed cross-sections cut at 15 DPA were stained with Aniline Blue for callose. (A) Image of the WT seed section. Note the strong callose signal (arrows) in the TCs in comparison with the buffer-only control (insert). The latter displayed no fluorescent signals in the TCs. (B–D) Images of the seed sections of RNAi line1, RNAi line5, and Co-S line1, respectively. Note that the callose signal in the TCs in these three lines was abolished entirely in comparison with the WT (A) and buffer-only control (insert in A). en, endosperm; isc, inner seed coat; tc, transfer cell, indicated by the white triangle. Scale bars=20 μm in A–D.

TCs are specialized transport cells containing invaginated wall ingrowths which could facilitate nutrient transport by amplifying the plasma membrane surface area (Offler *et al.*, 2003; Edwards *et al.*, 2010). In cotton seed, TCs are located at the inner surface of the inner seed coat (Ryser *et al.*, 1988; Ruan *et al.*, 1997) and are predicted to play a major role in channelling nutrients to the embryonic tissues (Ruan, 2013). Given the presence of callose and cellulose in cotton seed TCs (Pugh *et al.*, 2010), the abundance of callose and cellulose in the TCs was examined in 15 DPA seed using the callose-specific binding dye, Aniline Blue, and the cellulose detector, CFW, respectively. Figure 5A shows strong fluorescent signals of callose in seed coat TCs after staining with Aniline Blue, in comparison with that of buffer-only control (Fig. 5A insert). In contrast, the callose signal was reduced to an undetectable level in the TCs of the transgenic seeds (Fig. 5B–D).

**Fig. 6.**
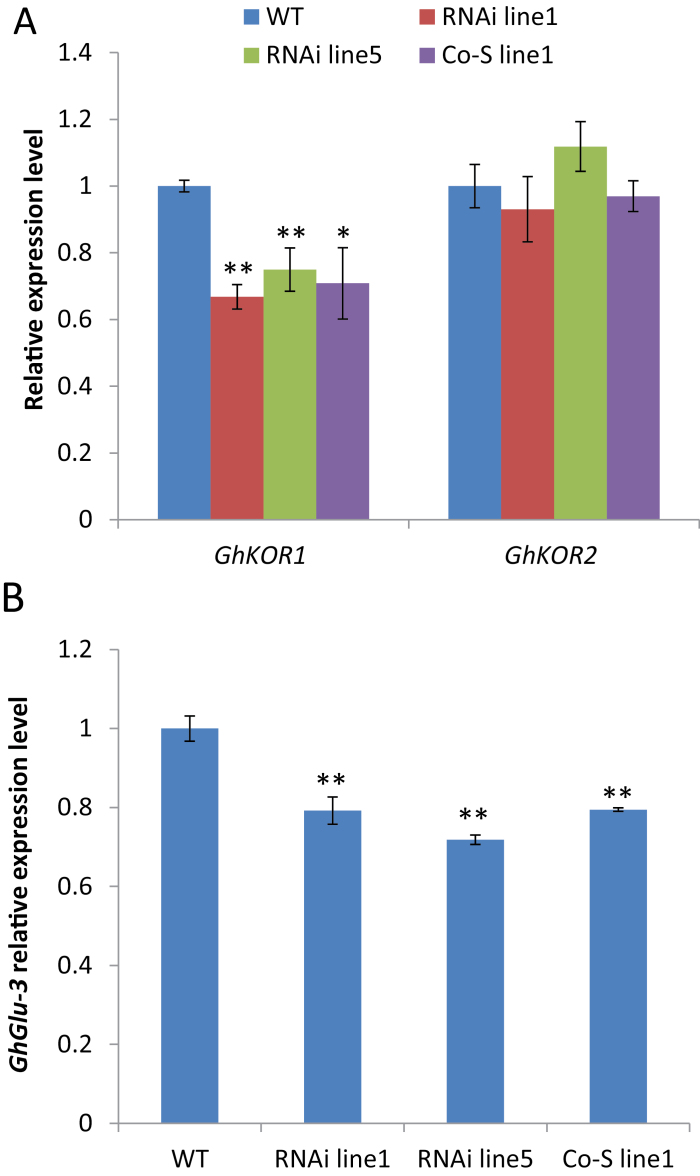
Transcript levels of *GhKOR1*, *GhKOR2*, and *GhGlu3* in 15 DPA seed coat of wild-type (WT), RNAi, and co-suppression (Co-S) transgenic cotton. (A) Transcript levels of *GhKOR1* and *GhKOR2* in 15 DPA seed coat. (B) Transcript levels of *GhGlu3* in 15 DPA seed coat. Note that the *GhGlu3* mRNA level was significantly down-regulated in the transgenic lines compared with the WT. * and ** indicate a significant difference from the WT by *t*-test with *P*-values of 0.05 and 0.01, respectively. (This figure is available in colour at *JXB* online.)

To investigate further the possible basis underlying the reduction of callose in transfer cells, two β-1,3-glucan synthase genes, *GhCas1* and *GhCas2*, and two endo-β-1,3-glucanase genes, *GhGlu3* and *GhGlu4*, which are highly expressed in cotton seed (according to the RNA-Seq results on developing cotton seed; unpublished data), were selected for measurement of their mRNA levels. As expected, the *GhKOR1* transcript level was reduced in 15 DAP seed coat (Fig. 6A) like that in the early stage (Fig. 2B). Interestingly, qRT-PCR analyses revealed that the transcript level of *GhGlu3* in 15 DPA seed coat was reduced by 21% in RNAi line1 and Co-S line1 and by 28% in RNAi line5, compared with that in the WT (Fig. 6B), while no or little changes were observed in *GhCas1*, *GhCas2*, and *GhGlu4* expression levels (Supplementary Fig. S5 at *JXB* online). Sequence alignment showed that *GhGlu3* cDNA shared high homology with the 562bp *GhKOR1* fragment used in the RNAi construct in some regions (Supplementary Fig. S3B). Similar to the functions of *GhKOR1* in cellulose (β-1,4-glucan) biosynthesis, *GhGlu3* could also be required in the biosynthesis of callose (β-1,3-glucan), and down-regulation of *GhGlu3* may contribute to the reduction of callose as indicated by Aniline Blue staining (Fig. 5).

Interestingly, although the *GhKOR1* expression level and crystalline cellulose content were reduced in the seed coat (Figs 2B, 3C, 6A), the fluorescence of cellulose in TCs, indicated by CFW staining, did not appear to exhibit any noticeable difference between the transgenic and WT seeds (Supplementary Fig. S6 at *JXB* online). A similar observation was previously made in the RNAi transgenic white spruce (*Picea glauca*), in which suppression of *KOR1* had no impact on cellulose abundance, as indicated by CFW staining, although cell wall structural glucose was reduced (Maloney *et al.*, 2012).

### Suppression of *GhKOR1* reduces cotton fibre development

**Fig. 7.**
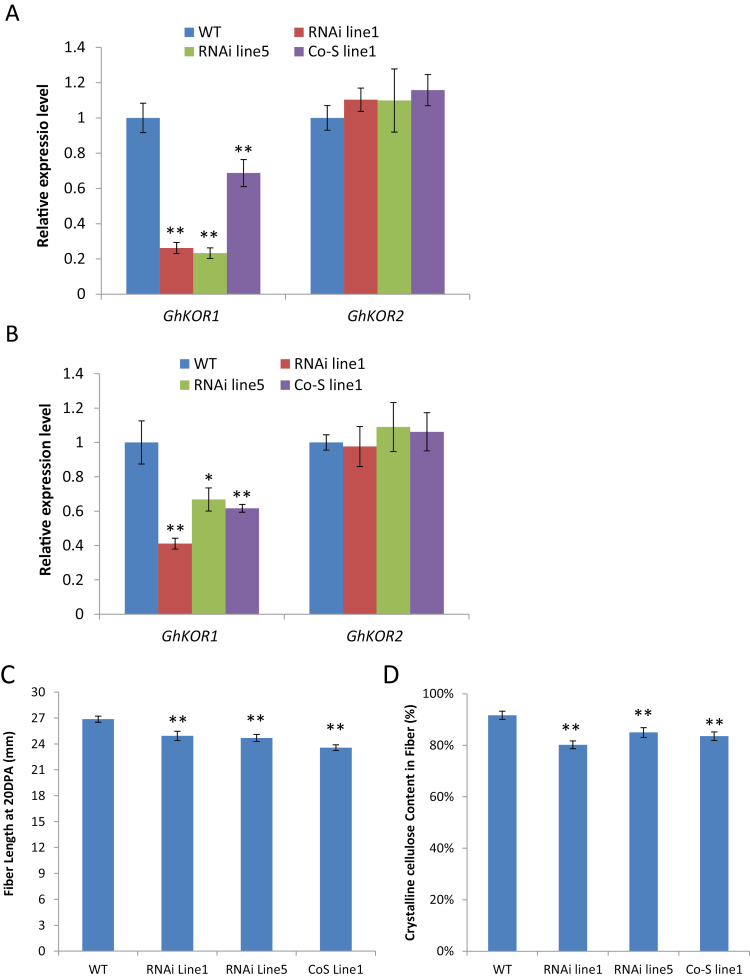
Suppression of *GhKOR1* reduced fibre length and crystalline cellulose content. (A and B) qRT-PCR results show a reduced *GhKOR1* mRNA level in 10 DPA and 20 DPA cotton fibre, respectively, of the three transgenic lines, with little or no impact on the *GhKOR2* mRNA level. (C) Fibre length at 20 DPA was reduced in the transgenic cotton as compared with that in the wild type. (D) Crystalline cellulose content in mature fibre of transgenic cotton was significantly reduced as compared with that in the wild type. * and ** indicate a significant difference from the wild type by *t*-test with *P*-values of 0.05 and 0.01, respectively. (This figure is available in colour at *JXB* online.)

Cotton fibres are single cells characterized by rapid elongation and cellulose synthesis in a sequential manner (Ruan, 2007). Given the potential role of KOR1 in these processes (Maloney *et al.*, 2012; Zhang *et al.*, 2012), it was further investigated whether silencing *GhKOR1* affects fibre growth at 10 and 20 DPA when fibre cells are at the peak of elongation and onset of cellulose synthesis, respectively (Ruan, 2007; Jiang *et al.*, 2012). The mRNA level of *GhKOR1* was significantly reduced in both 10 and 20 DAP cotton fibre, compared with that in the WT (Fig. 7A, B). Consistent with the expression pattern in seed coat and filial tissue, the transcript level of *GhKOR2* showed little or no changes in the 10 and 20 DAP fibres of the transgenic lines (Fig. 7A, B).

Measurement of fibre length at 20 DPA, when the fibre has fully elongated (Gipson and Ray, 1969), revealed a 7–12% reduction in fibre length as compared with the control (Fig. 7C). The crystalline cellulose content in mature fibre was reduced by 13, 7, and 9% in RNAi line1, RNAi line5, and Co-S line1, respectively, as compared with that of the WT (Fig. 7D). In addition, the mature fibre weight per seed was reduced by 25, 14, and 13%, respectively, in the transgenic plants as compared with the WT (Supplementary Fig. S7 at *JXB* online). It is likely that the reduced fibre weight resulted from both the reduced fibre length and reduced cellulose content. These findings demonstrate that suppression of *GhKOR1* in fibre development inhibits cell elongation and cellulose biosynthesis processes.

### Suppression of *GhKOR1* retards early seedling growth after germination

**Fig. 8.**
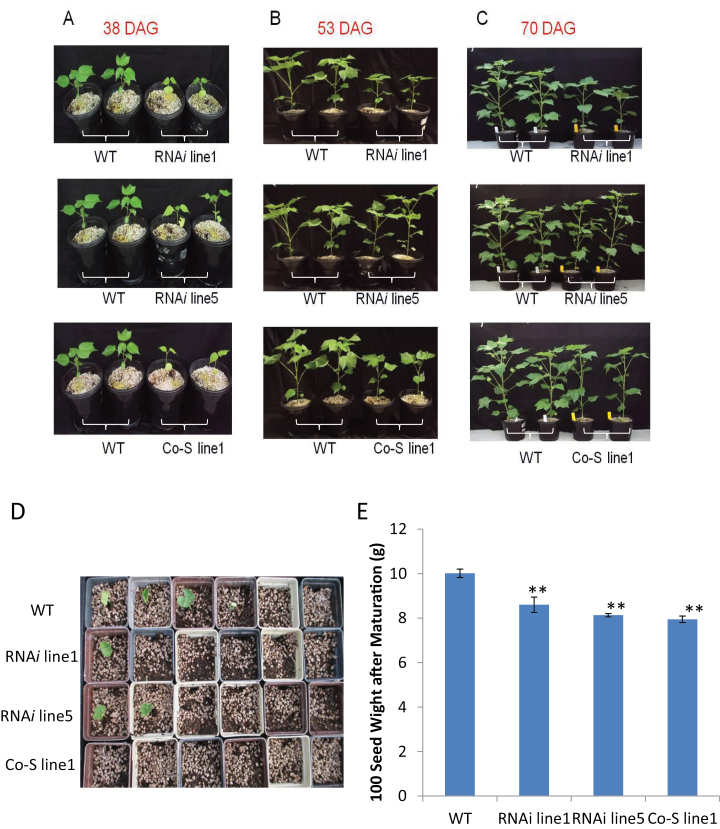
Reduced seedling growth in *GhKOR1*-down-regulated transgenic cotton. (A) The transgenic seedlings were significantly smaller and weaker than WT plants by 38 days after germination (DAG). (B) The transgenic plants were still smaller than the WT, but the difference appeared less obvious by 53 DAG than in the early stage as shown in (A). (C) By 70 DAG, just prior to flowering, the transgenic plants became phenotypically similar to the wild-type plants. (D) On the fifth day after the seeds were sown, among 15 seeds sown in each case, eight WT seedlings had emerged from the soil, while only two, five, and one seedling emerged in RNAi line1, RNAi line5, and Co-S line1, respectively. (E) After maturation, the 100 seed weight of transgenic lines was significantly reduced as compared with the WT. ** indicate a significant difference from the WT by *t*-test with a *P*-value of 0.01.

Finally, it was examined whether the disrupted endosperm cellularization and delayed embryo development in the transgenic seeds (Fig. 4) have any negative effects on early seedling vigour following germination. Figure 8 shows a clear slow-growing phenotype of the transgenic seedlings as compared with the WT. This phenotype was most evident at ~38 days after germination (DAG) but became less obvious at 53 DAG (Fig. 8A versus B). By 70 DAG, just prior to flowering, the transgenic plants no longer showed a visible growth difference from the WT plants, with each plant producing 9–10 true leaves (Fig. 8C). The reduced seedling growth seems to be associated with delayed emergence of the transgenic seedlings from the soil after sowing (Fig. 8D). Here, among 15 seeds sown for each of the WT and transgenic lines, eight seedlings emerged from the soil in the WT on the fifth day after sowing, whereas only two, five, and one seedling emerged in RNAi line1, RNAi line5, and Co-S line1, respectively. Interestingly, the degree of delayed seedling emergence phenotype in different transgenic lines correlates with the level of the filial tissue phenotype (Fig. 4). The reduced seedling vigour also correlates with the reduced mature seed weight (Fig. 8E).

Apart from being expressed in seed tissues, *GhKOR1* and *GhKOR2* were also expressed in vegetative tissues (Fig. 1). The transcript levels of these genes were thus measured in the root and above-ground tissue of the seedlings. The analyses revealed a significant reduction of the *GhKOR1*, but not the *GhKOR2*, mRNA level in both roots and above-ground tissues of the transgenic lines, compared with that in the WT (Supplementary Fig. S8 at *JXB* online).

## Discussion

### GhKOR1 is required for endosperm cellularization, embryo development, and early seedling vigour

Several lines of evidence have been provided in this study showing the requirement of GhKOR1 for seed development and early seedling vigour. Suppression of GhKOR1 disrupted endosperm cellularization and delayed embryo development (Figs 2, 4), resulting in reduced size and weight of filial tissue (Fig. 3) and delayed germination and reduced seedling vigour (Fig. 8). These findings are of significance for three reasons.

First, these observations show that GhKOR1 plays a critical role in seed development. This fills an important knowledge gap regarding the roles of KOR in plant development since research in this area has been focused exclusively on vegetative tissues thus far (Molhoj *et al.*, 2002; Somerville, 2006; Taylor, 2008). The present results represent an unprecedented example of the function of KOR in reproductive development.

Secondly, the finding that down-regulation of *GhKOR1* impaired endosperm cellularization sheds new light on the molecular basis of this cellular process, which is critical for seed development (Ruan, 2014). Endosperm cellularization is the cell wall formation phase, following a stage of free nuclear divisions without cytokinesis (syncytial phase) (Wang and Chien, 1957; Costa *et al.*, 2004; Ruan *et al.*, 2008). The process is known to be dependent on cellulose synthesis (Ruan *et al.*, 2008). Given this and the essential role of KOR1 in cellulose synthesis (Nicol *et al.*, 1998; Sato *et al.*, 2001; Szyjanowicz *et al.*, 2004) and cytokinesis demonstrated in a wide range of vegetative tissues, including *Arabidopsis* seedlings (Zuo *et al.*, 2000) and rice root (Zhang *et al.*, 2012), it is concluded that GhKOR1 is an essential player required for endosperm cellularization. Further studies on the potential linkage between GhKOR1 and the cellulose synthases/Sus complex could provide new insights into the regulatory mechanism underlying endosperm development in general and its cellularization in particular.

Thirdly, the phenotype of delayed embryo development in the *GhKOR1*-down-regulated seeds (Fig. 4) indicates that GhKOR1 is required for embryo development. This embryo phenotype may be attributed to disrupted endosperm cellularization as discussed above. The requirement of endosperm cellularization for proper embryo development has been well documented. In *Arabidopsis*, for example, failure of endosperm cellularization correlated with impaired embryo development in *fertilization independent seed 2* (*fis2*) and *endosperm defective 1* (*ede1*) *Arabidopsis* mutants (Hehenberger *et al.*, 2012). Research on interploidy crosses (Scott *et al.*, 1998) and cotton transgenic work (Ruan *et al.*, 2008) also provide clear evidence that endosperm cellularization is critical for embryo development. Alternatively, GhKOR1 may also be directly involved in early embryo development which is characterized by intensive cell division. To this end, KOR has been shown to play a role in cell division (Zuo *et al.*, 2000; Krupkova and Schmulling, 2009; Zhang *et al.*, 2012). It is also of significance to note that the seedlings germinated from the *GhKOR1*-suppressed transgenic seed grew noticeably more slowly than their WT counterparts (Fig. 8). Given the compromised seed development evidenced by the impaired endosperm cellularization and delayed embryo development, the slow seedling growth phenotype is most probably due to reduced seed quality or vigour. Interestingly, the seedling phenotype is restricted to the first 40 DAG or so, since the transgenic seedlings were able to catch up later and became phenotypically identical to WT plants by flowering time (Fig. 8). These observations reinforce the view that the slow growth of the seedling relates more to the reduced seed vigour than to the potential effects of reduced GhKOR1 on the seedling *per se*. However, the latter possibility cannot be excluded since *GhKOR1* was also down-regulated in vegetative tissues of seedlings (Supplementary Fig. S8 at *JXB* online). Overall, the data provide a remarkable example of the role of KOR in seed development and early seedling vigour.

### GhKOR1 is essential for callose deposition in transfer cells and involved in fibre development of seed coat

Developing cotton seed is unique in that its maternal seed coat develops two specialized cells at its opposite sides: TCs at the innermost cell layer and fibre cells on the outermost epidermis (Ruan, 2005). Cotton seed TCs are enriched in callose and, to a lesser extent, cellulose (Pugh *et al.*, 2010). The present analyses show that suppression of *GhKOR1* in 12 and 15 DPA seed coat (Figs 2B, 6A) reduced the crystalline cellulose content significantly in 20 DPA seed coat of transgenic lines (Fig. 3C). This result is in agreement with previous studies showing that KOR is required for cellulose synthesis (Nicol *et al.*, 1998; Sato *et al.*, 2001; Szyjanowicz *et al.*, 2004). Another interesting finding is that callose deposition was abolished in the TC of 15 DAP seed coat (Fig. 5). The synthesis of both callose and cellulose uses UDP-glucose as the substrate, with the former polymerized in β-1,3- and the latter in β-1,4-linkages. Given the similarity in the biochemistry for the synthesis of the two sugar polymers, one may envisage similar regulatory mechanisms for their production. Surprisingly, despite extensive evidence on the requirement of KOR for cellulose synthesis (Nicol *et al.*, 1998; Sato *et al.*, 2001; Szyjanowicz *et al.*, 2004), there have been no reports on its potential role in callose synthesis. The present finding of the absense of callose in TCs in *GhKOR1*-suppressed seed (Fig. 5) provides compelling evidence that GhKOR1 is a major player for callose deposition in TCs of cotton seed coat. However, *GhGlu3*, one highly expressed gene in cotton seed, was found to be significantly down-regulated in 15 DPA seed coat (Fig. 6B), probably as a result of the homology-dependent co-suppression with *GhKOR1* (Supplementary Fig. S3B at *JXB* online). As an endo-β-1,3-glucanase, GhGlu3 could play a similar role in callose biosynthesis to that which GhKOR1 plays in cellulose biosynthesis (see the Introduction). Thus reduced expression of *GhGlu3* may also contribute to the abolishment of callose in TC.

It remains unclear whether the abolishment of callose (Fig. 5) affects the function of the TCs, since no significant changes of TC morphology were observed in the *GhKOR1*-suppressed seed (Figs 4, 5; Supplementary Fig. S6 at *JXB* online). Regardless, any potential negative effect on TC function is unlikely to be resposible for the disrupted endosperm cellularization and delayed embryo development (Fig. 4). This assertion is based on the fact that the filial tissue phenotype was evident at 10 DPA (Fig. 4) before the formation of TCs (Pugh *et al.*, 2010).

Cotton fibres are single-celled hairs undergoing rapid elongation driven by cell turgor (Ruan *et al.*, 1996, 2001) followed by massive deposition of cellulose (Ruan, 2005). Consistent with the role of KOR in these processes as shown in other systems (e.g. Nicol *et al.*, 1998; Sato *et al.*, 2001; Szyjanowicz *et al.*, 2004; Taylor, 2008), GhKOR1 expression increased sharply in WT fibres at 10 DPA and remained high up to 25 DPA (Fig. 1B) at the onset of fibre elongation and the peak time of cellulose synthesis, respectively (Ruan, 2005, 2007). Down-regulation of *GhKOR1* in fibres reduced fibre length and crystalline cellulose content (Fig. 7), demonstrating a role for GhKOR1 in cotton fibre development, which is under complex control involving numerous proteins and signalling pathways (Jiang *et al.*, 2012; Ruan, 2013; Wang *et al.*, 2014)

**Fig. 9.**
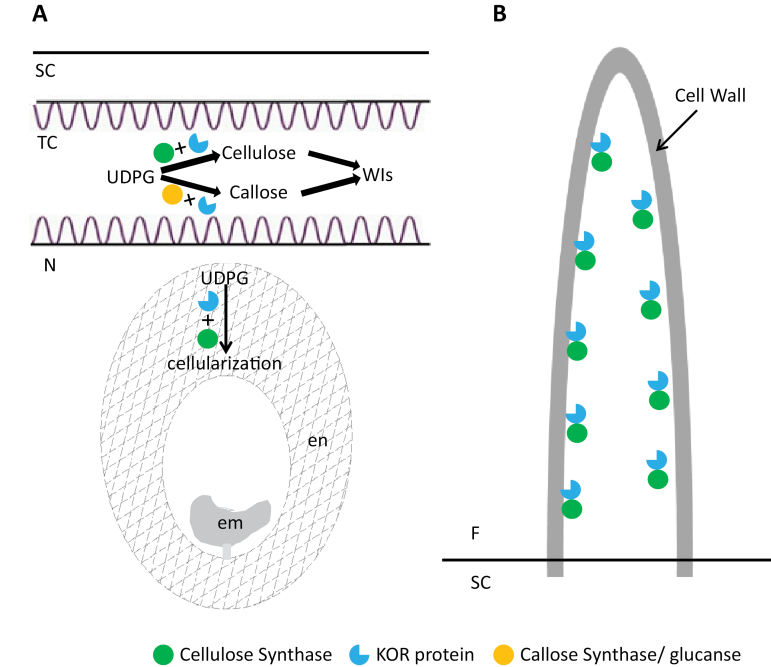
A schematic model of the roles that GhKOR1 may play in endosperm cellularization, wall ingrowths of transfer cells (A), and fibre cell development (B). (A) Cotton seed TCs are enriched in callose and cellulose. Suppression of *GhKOR1* in the seed coat slightly reduced the crystalline cellulose content in 20 DPA seed coat butt abolished the callose content in 15 DPA TCs, which might compromise TC function. Endosperm cellularization is a process that is dependent on cellulose biosynthesis which may require expression of GhKOR1 based on the observation that down-regulation of *GhKOR1* disrupted endosperm development and delayed embryo development. The compromised seed development reduced the seedling vigour of the transgenic plants. (B) Cotton fibre undergoes rapid cell elongation at 10–15 DPA and massive cell wall cellulose biosynthesis at 20 DPA onwards. Both processes may be dependent on the concerted action of cellulase synthase and GhKOR1 since suppression of *GhKOR1* shortened fibres and reduced their cellulose content. em, embryo; en, endosperm; F, fibre; N, nucellus; SC, seed coat; TC, transfer cell; WIs, wall ingrowths.

In conclusion, this study provided novel evidence that expression of *GhKOR1* is essential for endosperm cellularization, embryo development, and seedling vigour. The analyses also showed that GhKOR1 plays a major role in callose deposition in seed coat TCs and is involved in cotton fibre elongation and cellulose synthesis. Figure 9 shows a schematic model of how GhKOR1 may function in cotton seed and fibre, potentially through interplay with proteins involved in biosynthesis of cellulose and callose. Overall, the data demonstrated important roles of GhKOR1 in the development of both filial and maternal seed tissues.

## Supplementary data

Supplementary data are available at *JXB* online.

Figure S1. A schematic map of the structures of the *GhKOR1* and *AtKOR1* genes.

Figure S2. Alignment of eight different plant membrane-bound endo-1,4-β-glucanase protein sequences.

Figure S3. The alignment between the RNAi fragment of *GhKOR1* used in the RNAi construct, and *GhKOR2* cDNA and *GhGlu3* cDNA sequences.

Figure S4. Fresh and dry weights of the 20 DPA seed coat in *GhKOR1*-down-regulated transgenic cotton in comparison with the wild type.

Figure S5. Transcript levels of *GhCas1*, *GhCas2*, and *GhGlu4* in 15 DPA seed coat of wild-type, RNAi, and co-suppression (Co-S) transgenic cotton.

Figure S6. Comparison of aniline blue-labelled cellulose signals in 15 DPA seed transfer cells between wild-type and *GhKOR1*-down-regulated transgenic cotton.

Figure S7. Reduced fibre weight in *GhKOR1*-down-regulated transgenic cotton.

Figure S8. Transcript levels of *GhKOR1* and *GhKOR2* in root and above-ground tissue of wild-type, RNAi, and co-suppression (Co-S) transgenic cotton.

Table S1. Primers used for PCR and RT-PCR analyses.

Supplementary Data
