# Supplementary material for: Down-regulation of the cotton endo-1,4-β-glucanase gene KOR1 disrupts endosperm cellularization, delays embryo development, and reduces early seedling vigour
Source: J Exp Bot. 2015 Mar 24;66(11):3071–83. doi: 10.1093/jxb/erv111 (PMC4449532; doi:10.1093/jxb/erv111)
Supplement: Supplementary Data [file supp_66_11_3071__index.html]

Down-regulation of the cotton endo-1,4-β-glucanase gene KOR1 disrupts endosperm cellularization, delays embryo development, and reduces early seedling vigour — Down-regulation of the cotton endo-1,4-β-glucanase gene KOR1 disrupts endosperm cellularization, delays embryo development, and reduces early seedling vigour — Supplementary Data 

# Down-regulation of the cotton endo-1,4-β-glucanase gene *KOR1* disrupts endosperm cellularization, delays embryo development, and reduces early seedling vigour

## Supplementary Data

Data files

**Files in this Data Supplement:**

- Supplementary Data - Supplementary Data
- Supplementary Data - Supplementary Data
