# Supplementary material for: Down-regulation of the cotton endo-1,4-β-glucanase gene KOR1 disrupts endosperm cellularization, delays embryo development, and reduces early seedling vigour
Source: J Exp Bot. 2015 Mar 24;66(11):3071–83. doi: 10.1093/jxb/erv111 (PMC4449532; doi:10.1093/jxb/erv111)
Supplement: Supplementary Data [file supp_erv111_jexbot144881_file001.pdf]

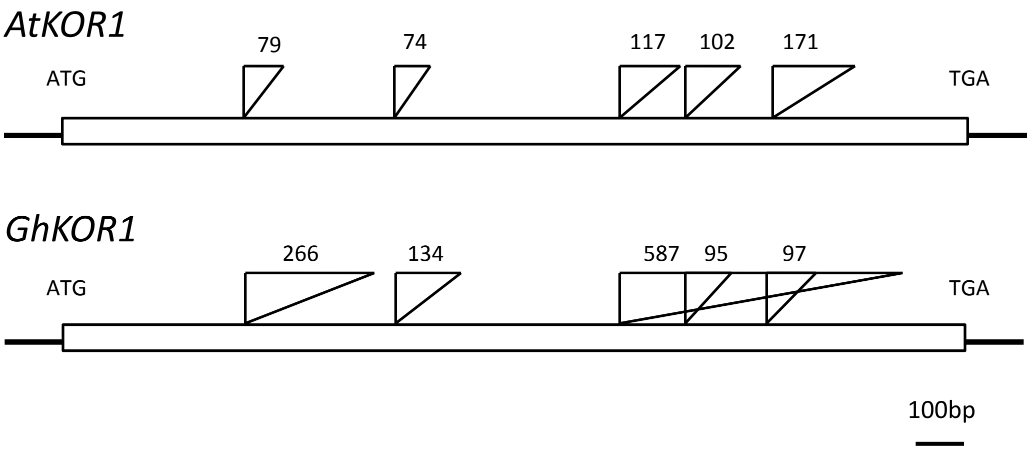

Supplemental figure S1. A schematic map of the gene structures of the *GhKOR1* and *AtKOR1* genes. Triangles indicate the location and size of the introns.

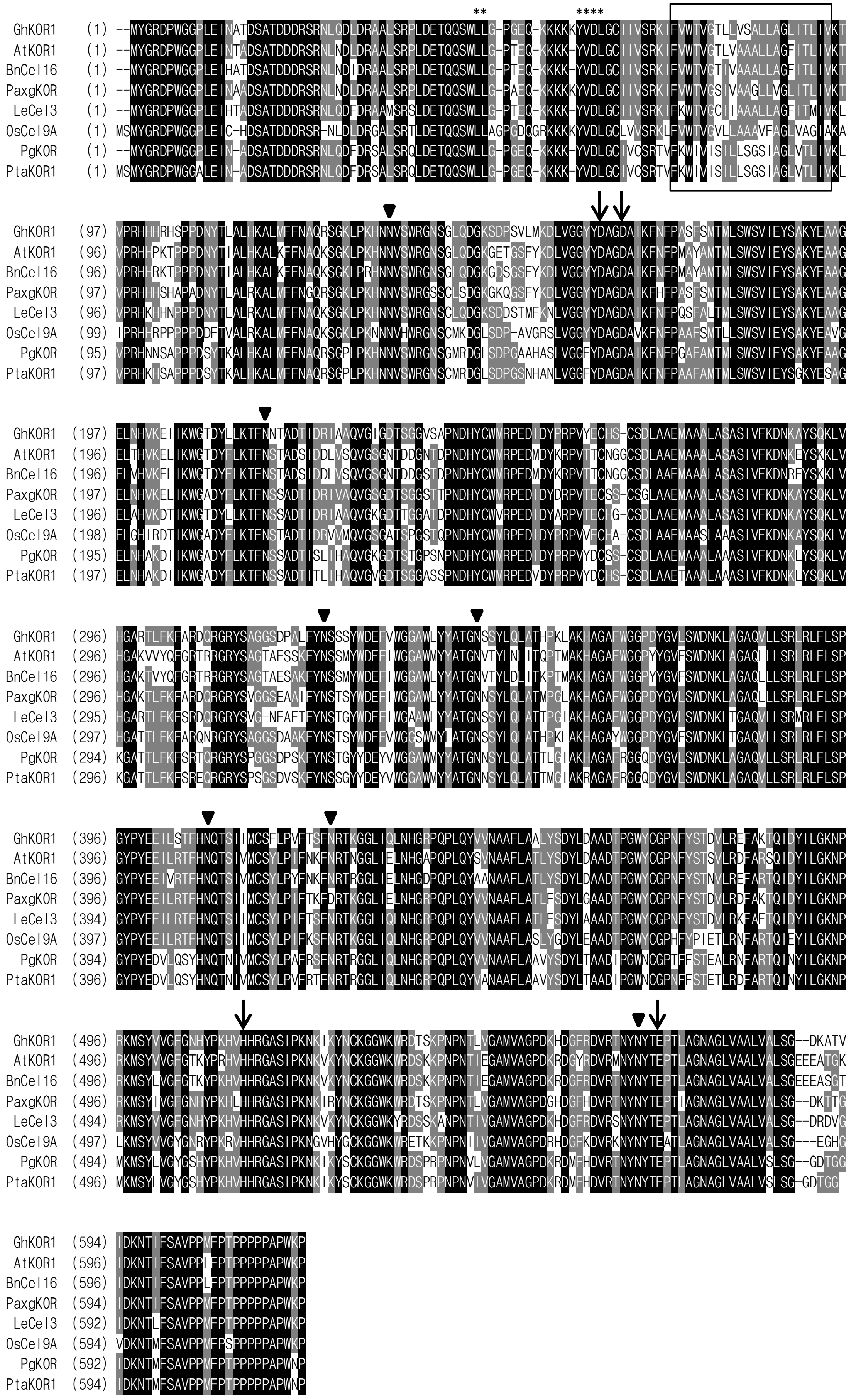

** ****
GhKOR1 (1) --MYGRDPWGGPLEINATDSATDDDRSRNLQDLDRAALSRPLDETQQSWLLG-PGEQ-KKKKKYVDLGCIIVSRKIFVWTVGTLLVSALLAGLITLIVKT
AtKOR1 (1) --MYGRDPWGGPLEINTADSATDDDRSRNLNDLDRAALSRPLDETQQSWLLG-PTEQ-KKKK-YVDLGCIIVSRKIFVWTVGTLVAAALLAGFITLIVKT
BnCel16 (1) --MYGRDPWGGPLEIHATDSATDDDRSRNLNDIDRAALSRPLDETQQSWLLG-PTEQ-KKKK-YVDLGCIIVSRKIFVWTVGTIVAAALLAGFITLIVKT
PaxgKOR (1) --MYGRDPWGGPLEINAADSATDDDRSRNLNDLDRAALSRPLDETQQSWLLG-PAEQ-KKKKKYVDLGCIIVSRKIFVWTVGSIVAAGLLVGLITLIVKT
LeCel3 (1) --MYGRDPWGGPLEIHTADSATDDDRSRNLQDFDRAAMSRSLDETQQSWLLG-PTEQ-KKKK-YVDLGCIIVSRKIFKWTVGCIIAAALLAGFITMIVKL
OsCel9A (1) MSMYGRDPWGGPLEIC-HDSATDDDRSR-NLDLDRGALSRTLDETQQSWLLAGPGDQGRKKKKYVDLGCLVVSRKLFVWTVGVLLAAAVFAGLVAGIAKA
PgKOR (1) --MYGRDPWGGPLEIN-ADSATDDDRSRNLQDFDRSALSRQLDETQQSWLLG-PGEQ-KKKK-YVDLGCIVCSRTVFKWIVISILLSGSIAGLVTLIVKL
PtaKOR1 (1) MSMYGRDPWGGALEIN-ADSATDDDRSRNLQDFDRSALSRQLDETQQSWLLG-PGEQ-KKKK-YVDLGCIVCSRTVFKWIVISILLSGSIAGLVTLIVKL
GhKOR1 (97) VPRHHHRHSPPDNYTLALHKALMFFNAQRSGKLPKHNNVSWRGNSGLQDGKSDPSVLMKDLVGGYYDAGDAIKFNFPASFSMTMLSWSVIEYSAKYEAAG
AtKOR1 (96) VPRHHPKTPPPDNYTIALHKALKFFNAQKSGKLPKHNNVSWRGNSGLQDGKGETGSFYKDLVGGYYDAGDAIKFNFPMAYAMTMLSWSVIEYSAKYEAAG
BnCel16 (96) VPRHHRKTPPPDNYTIALHKALKFFNAQKSGKLPRHNNVSWRGNSGLQDGKGDSGSFYKDLVGGYYDAGDAIKFNFPMAYAMTMLSWSVIEYSAKYEAAG
PaxgKOR (97) VPRHHHSHAPADNYTLALRKALMFFNGQRSGKLPKHNNVSWRGSSCLSDGKGKQGSFYKDLVGGYYDAGDAIKFHFPASFSMTMLSWSVIEYSAKYEAAG
LeCel3 (96) VPRHKHHNPPPDNYTLALRKALMFFNAQKSGKLPKHNNVSWRGNSCLQDGKSDDSTMFKNLVGGYYDAGDAIKFNFPQSFALTMLSWSVIEYSAKYEAAG
OsCel9A (99) IPRHHRPPPPPDDFTVALRKALMFFNAQKSGKLPKNNNVHWRGNSCMKDGLSDP-AVGRSLVGGYYDAGDAVKFNFPAAFSMTLLSWSVIEYSAKYEAVG
PgKOR (95) VPRHNNSAPPPDSYTKALHKALMFFNAQRSGPLPKHNNVSWRGNSGMRDGLSDPGAAHASLVGGFYDAGDAIKFNFPGAFAMTMLSWSVIEYSAKYEAAG
PtaKOR1 (97) VPRHKHSAPPPDSYTKALHKALMFFNAQRSGPLPKHNNVSWRGNSCMRDGLSDPGSNHANLVGGFYDAGDAIKFNFPAAFAMTMLSWSVIEYSGKYESAG
GhKOR1 (197) ELNHVKEIIKWGTDYLLKTFNNTADTIDRIAAQVGIGDTSGGVSAPNDHYCWMRPEDIDYPRPVYECHS-CSDLAAEMAALASASIVFKDNKAYSQKLV
AtKOR1 (196) ELTHVKELIKWGTDYFLKTFNSTADSIDDLVSQVGSGNTDDGNTDPNDHYCWMRPEDMDYKRPVTTCNGGCSDLAAEMAALASASIVFKDNKEYSKKLV
BnCel16 (196) ELVHVKELIKWGTDYFLKTFNSTADSIDDLVSQVGSGNTDDGSTDPNDHYCWMRPEDMDYKRPVTTCNGGCSDLAAEMAALASASIVFKDNREYSKKLV
PaxgKOR (197) ELNHVKELIKWGADYFLKTFNSSADTIDRIVAQVGSGDTSGGSTTPNDHYCWMRPEDIDYDRPVTECSS-CSGLAAEMAALASASIVFKDNKAYSQKLV
LeCel3 (196) ELAHVKDTIKWGTDYLLKTFNSSADTIDRIAAQVGKGDTTGGATDPNDHYCWVRPEDIDYARPVTECHG-CSDLAAEMAALASASIVFKDNKAYSQKLV
OsCel9A (198) ELGHIRDTIKWGADYFLKTFNSTADTIDRVVMQVGSGATSPGSTQPNDHYCWMRPEDIDYPRPVVECHA-CSDLAAEMASLAAASIVFKDNKAYSQKLV
PgKOR (195) ELNHAKDIIKWGADYFLKTFNSSADTISLIHAQVGKGDTSTGPSNPNDHYCWMRPEDIDYPRPVYDCSS-CSDLAAEMAALAAASIVFKDNKLYSQKLV
PtaKOR1 (197) ELNHAKDIIKWGADYFLKTFNSSADTITLIHAQVGVGDTSGGASSPNDHYCWMRPEDVDYPRPVYDCHS-CSDLAAETAALAAASIVFKDNKLYSQKLV
GhKOR1 (296) HGARTLFKFARDQRGRYSAGGSDPALFYNSSSYWDEFVWGGAWLYYATGNSSYLQLATHPKLAKHAGAFWGGPDYGVLSWDNKLAGAQVLLSRLRLFLSP
AtKOR1 (296) HGAKVVYQFGRTRRGRYSAGTAESSKFYNSSMYWDEFIWGGAWMYYATGNVTYLNLITQPTMAKHAGAFWGGPYYGVFSWDNKLAGAQLLLSRLRLFLSP
BnCel16 (296) HGAKTVYQFGRTRRGRYSAGTAESAKFYNSSMYWDEFIWGGAWLYYATGNVTYLDLITKPTMAKHAGAFWGGPYYGVFSWDNKLAGAQLLLSRLRLFLSP
PaxgKOR (296) HGAKTLFKFARDQRGRYSVGGSEAAIFYNSTSYWDEFIWGGAWLYYATGNNSYLQLATMPGLAKHAGAFWGGPDYGVLSWDNKLAGAQLLLSRLRLFLSP
LeCel3 (295) HGARTLFKFSRDQRGRYSVG-NEAETFYNSTGYWDEFIWGAAWLYYATGNSSYLQLATTPGIAKHAGAFWGGPDYGVLSWDNKLTGAQVLLSRMRLFLSP
OsCel9A (297) HGATTLFKFARQNRGRYSAGGSDAAKFYNSTSYWDEFVWGGSWMYLATGNSSYLQLATHPKLAKHAGAYWGGPDYGVFSWDNKLTGAQVLLSRLRLFLSP
PgKOR (294) KGATTLFKFSRTQRGRYSPGGSDPSKFYNSTGYYDEYVWGGAWMYYATGNNSYLQLATTLGIAKHAGAFRGGQDYGVLSWDNKLAGAQVLLSRLRLFLSP
PtaKOR1 (296) KGATTLFKFSREQRGRYSPSGSDVSKFYNSSGYYDEYVWGGAWMYYATGNNSYLQLATTMGIAKRAGAFRGGQDYGVLSWDNKLAGAQVLLSRLRLFLSP
GhKOR1 (396) GYPYEEILSTFHNQTSIIMCSFLPVFTSFNRTKGGLIQLNHGRPQPLQYVVNAAFLAALYSDYLDAADTPGWYCGPNFYSTDVLREFAKTQIDYILGKNP
AtKOR1 (396) GYPYEEILRTFHNQTSIVMCSYLPIFNKFNRTNGGLIELNHGAPQPLQYSVNAAFLATLYSDYLDAADTPGWYCGPNFYSTSVLRDFARSQIDYILGKNP
BnCel16 (396) GYPYEEIVRTFHNQTSIVMCSYLPYFNKFNRTRGGLIELNHGDPQPLQYAANAAFLATLYSDYLDAADTPGWYCGPNFYSTNVLREFARTQIDYILGKNP
PaxgKOR (396) GYPYEEILRTFHNQTSIIMCSYLPIFTKFDRTKGGLIELNHGRPQPLQYVVNAAFLATLFSDYLGAADTPGWYCGPNFYSTDVLRDFAKTQIDYILGKNP
LeCel3 (394) GYPYEEILRTFHNQTSIIMCSYLPIFTSFNRTKGGLIQLNHGRPQPLQYVVNAAFLATLFSDYLAAADTPGWYCGPNFYSTDVLRKFAETQIDYILGKNP
OsCel9A (397) GYPYEEILRTFHNQTSIIMCSYLPIFKSFNRTKGGLIQLNHGRPQPLQYVVNAAFLASLYGDYLEAADTPGWYCGPHFYPIETLRNFARTQIEYILGKNP
PgKOR (394) GYPYEDVLQSYHNQTNIVMCSYLPAFRSFNRTRGGLIQLNHGRPQPLQYVVNAAFLAAVYSDYLTAADIPGWNCGPTFFSTEALRNFARTQINYILGKNP
PtaKOR1 (396) GYPYEDVLQSYHNQTNIVMCSYLPVFRTFNRTRGGLIQLNHGRPQPLQYVANAAFLAAVYSDYLTAADIPGWNCGPNFFSTETLRDFARTQINYILGKNP
GhKOR1 (496) RKMSYVVGFGNHYPKHVHHRGASIPKNKIKYNCKGGWKWRDTSKPNPNTLVGAMVAGPDKHDGFRDVRTNYNYTEPTLAGNAGLVAALVALSG--DKATV
AtKOR1 (496) RKMSYVVGFGTKYPRHVHHRGASIPKNKVKYNCKGGWKWRDSKKPNPNTIEGAMVAGPDKRDGYRDVRMNYNYTEPTLAGNAGLVAALVALSGEEEATGK
BnCel16 (496) RKMSYLVGFGTKYPKHVHHRGASIPKNKVKYNCKGGWKWRDSKKPNPNTIEGAMVAGPDKRDGFRDVRTNYNYTEPTLAGNAGLVAALVALSGEEEASGT
PaxgKOR (496) RKMSYIVGFGNHYPKHLHHRGASIPKNKIRYNCKGGWKWRDTSKPNPNTLVGAMVAGPDGHDGFHDVRTNYNYTEPTIAGNAGLVAALVALSG--DKTTG
LeCel3 (494) RKMSYVVGFGNHYPKHVHHRGASIPKNKVKYNCKGGWKYRDSSKANPNTIVGAMVAGPDKHDGFRDVRSNYNYTEPTLAGNAGLVAALVALSG--DRDVG
OsCel9A (497) LKMSYVVGYGNRYPKRVHHRGASIPKNGVHYGCKGGWKWRETKKPNPNIIVGAMVAGPDRHDGFKDVRKNYNYTEATLAGNAGLVAALVALSG---EGHG
PgKOR (494) MKMSYLVGYGSHYPKHVHHRGASIPKNKIKYSCKGGWKWRDSPRPNPNVLVGAMVAGPDKRDMFHDVRTNYNYTEPTLAGNAGLVAALVSLSG--GDTGG
PtaKOR1 (496) MKMSYLVGYGSHYPKHVHHRGASIPKNKIKYSCKGGWKWRDSPRPNPNVIVGAMVAGPDKRDMFHDVRTNYNYTEPTLAGNAGLVAALVSLSG-GDTGG
GhKOR1 (594) IDKNTIFSAVPPMFPTPPPPPAPWKP
AtKOR1 (596) IDKNTIFSAVPPLFPTPPPPPAPWKP
BnCel16 (596) IDKNTIFSAVPPLFPTPPPPPAPWKP
PaxgKOR (594) IDKNTIFSAVPPMFPTPPPPPAPWKP
LeCel3 (592) IDKNTLFSAVPPMFPTPPPPPAPWKP
OsCel9A (594) VDKNTMFSAVPPMFPSPPPPPAPWKP
PgKOR (592) IDKNTMFSAVPPMFPTPPPPPAPWNP
PtaKOR1 (594) IDKNTMFSAVPPMFPTPPPPPAPWNP

Supplemental figure S2. Alignment of eight different plant membrane-bound endo-1,4-b-glucanase protein sequences. Identical amino acids were shaded in black. Stars denote polarized targeting signals. Twenty-two boxed amino acids indicate the predicted transmembrane domains. Arrowheads show the predicted glycosylation sites; Arrows denote the residues essential for catalytic activity. *GhKOR1, Gossypium hirstum*, AAS87601; *AtKOR1, Arabidopsis thaliana*, NP_199783(At5g49720); *BnCel16, Brassica napus*, CAB51903; *Pa x gKOR, Populus alba x grandidentata*, ADB82903; *LeCel3, Lycopersicon esculentum,* AAC49704; *OsCel9A, Oryza sativa*, BAF37260; *PgKOR, Picea glauca*, AEL88496; *PtrKOR1, Populus trichocarpa*, AAS45400.

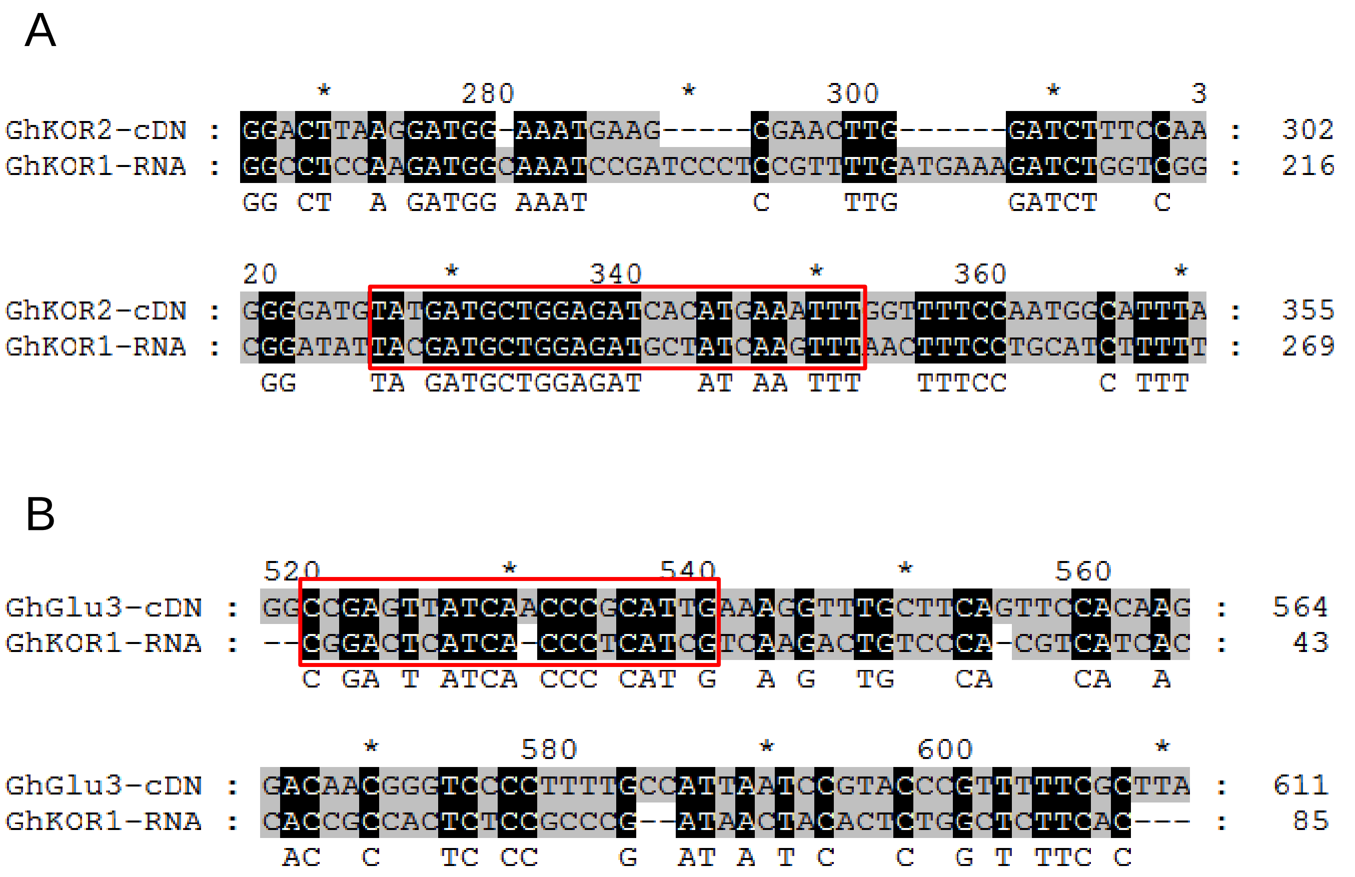

Supplemental Figure S3. The alignment between the RNAi fragment of *GhKOR1* used in the RNAi construct and *GhKOR2* cDNA, *GhGlu3* cDNA sequences. The red boxed sequences indicate regions with high sequence homology which may cause co-suppression.

(A) The alignment between the RNAi fragment of *GhKOR1* and *GhKOR2* cDNA sequences;

(B) The alignment between the RNAi fragment of *GhKOR1* and *GhGlu3* cDNA sequences

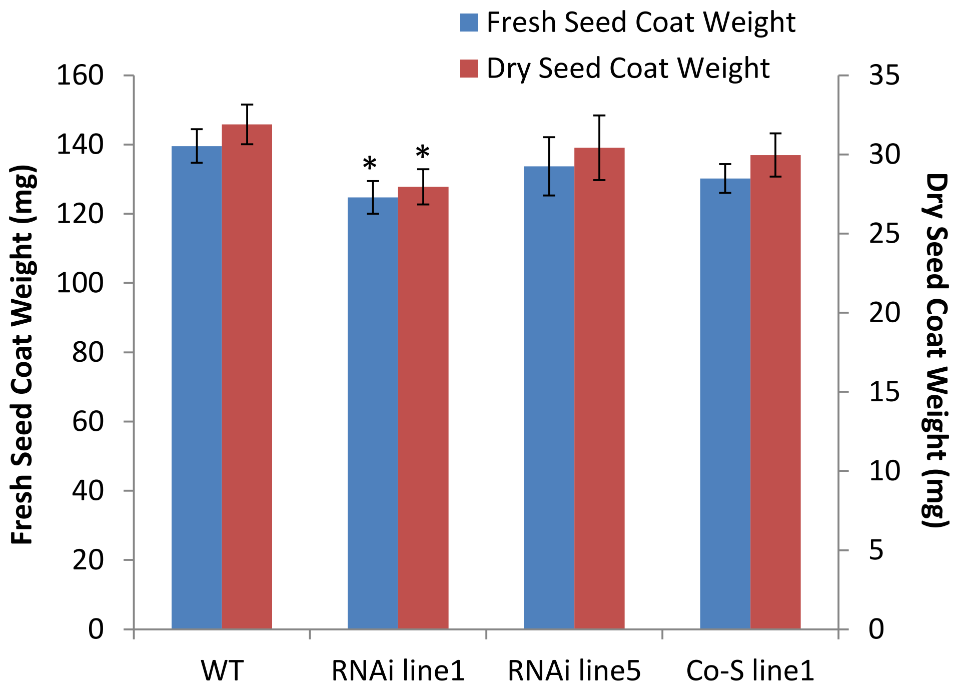

Supplemental Figure S4. Fresh and dry weight of the 20 DPA seed coat in *GhKOR1* down-regulated transgenic cotton in comparison with that of wild-type.
Except for RNAi line1, the fresh and dry weight of 20-d seed coat remained unchanged in the transgenic cotton compared to that in WT. * indicate a significant difference to wild type in *t*-test with *P*-value of 0.05. At least three biological replicates were used for each case.

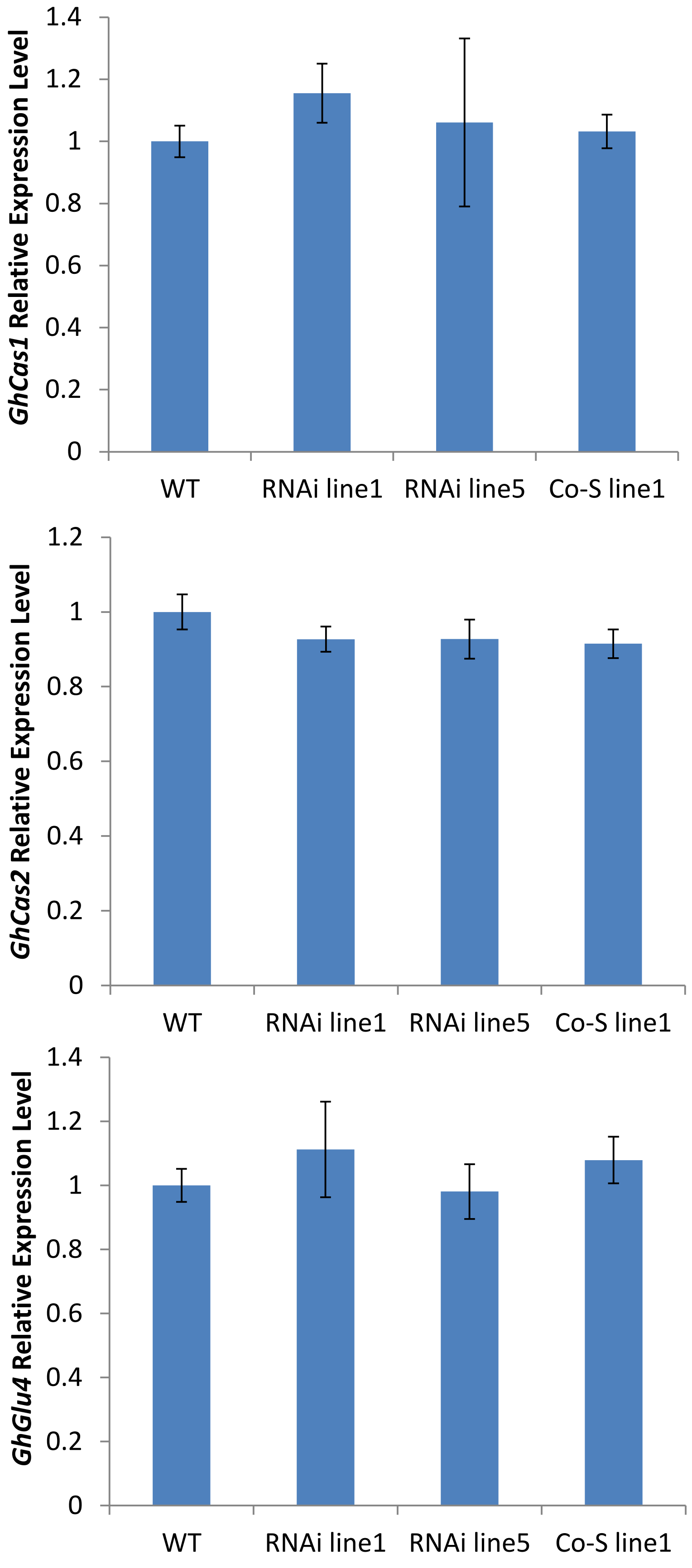
GhCas1 Relative Expression Level
1.4
1.2
1
0.8
0.6
0.4
0.2
0
WT
RNAi line1
RNAi line5
Co-S line1
GhCas2 Relative Expression Level
1.2
1
0.8
0.6
0.4
0.2
0
WT
RNAi line1
RNAi line5
Co-S line1
GhGlu4 Relative Expression Level
1.4
1.2
1
0.8
0.6
0.4
0.2
0
WT
RNAi line1
RNAi line5
Co-S line1

Supplemental Figure S5. **Transcript levels of *GhCas1*, *GhCas2* and *GhGlu4* in 15 DPA seed coat of wide type (WT), RNAi and co-suppression (Co-S) transgenic cotton.**

(A)Transcript levels of *GhCas1* in 15 DPA seed coat.

(B) Transcript levels of *GhCas2* in 15 DPA seed coat.

(C) Transcript levels of *GhGlu4* in 15 DPA seed coat.

No significant difference was detected in the transcript level of these three callose metabolism genes in 15 DPA seed coat.

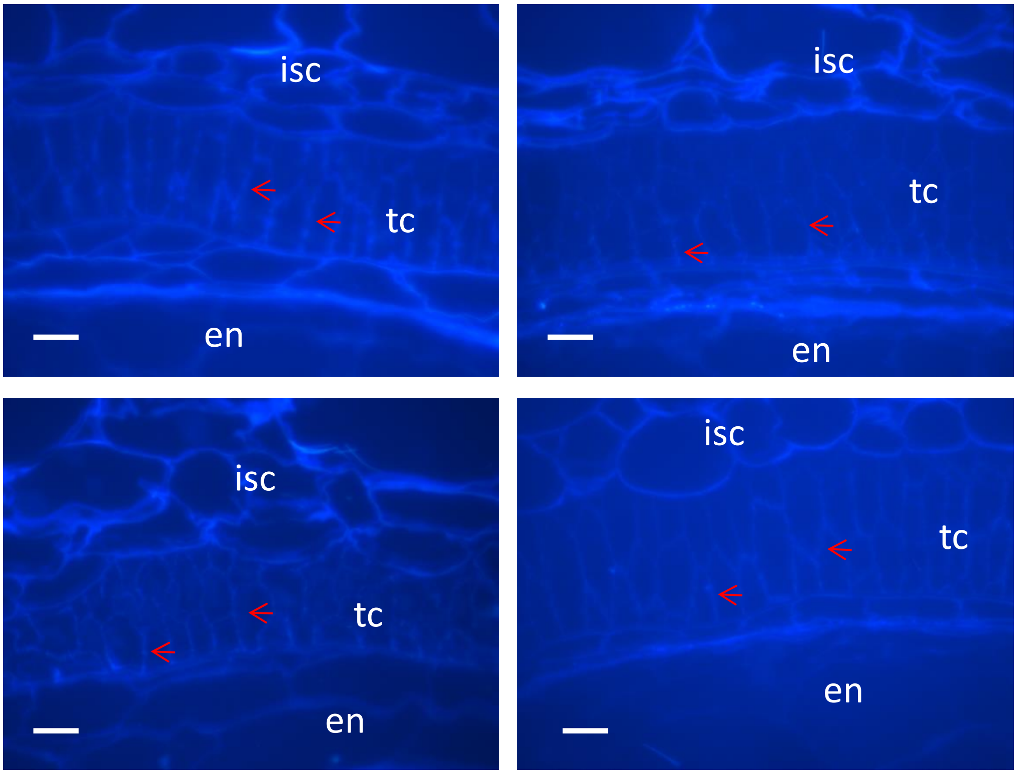

Supplemental Figure S6. Comparison of aniline blue – labelled cellulose signals in 15DPA seed transfer cells (TCs) between WT and *GhKOR1* down-regulated transgenic cotton. Images are micrographs of 2µm cross sections of 15DPA resin embedded seeds stained with Calcofluor White. (A) Image of the WT 15DPA cotton seed section; (B to D) Images of the RNAi line1, RNAi line5 and Co-S line1 15DPA cotton seed section, respectively. No significant difference of the cellulose signals could be detected in the TCs between the transgenic lines and the WT, which showed similar flange morphology of the TCs (red arrows). Three biological replicates were observed for each line with similar results obtained. Representative images were provided.
en, endosperm; isc, inner seed coat; tc, transfer cell. Bars = 50 µm in A to D.

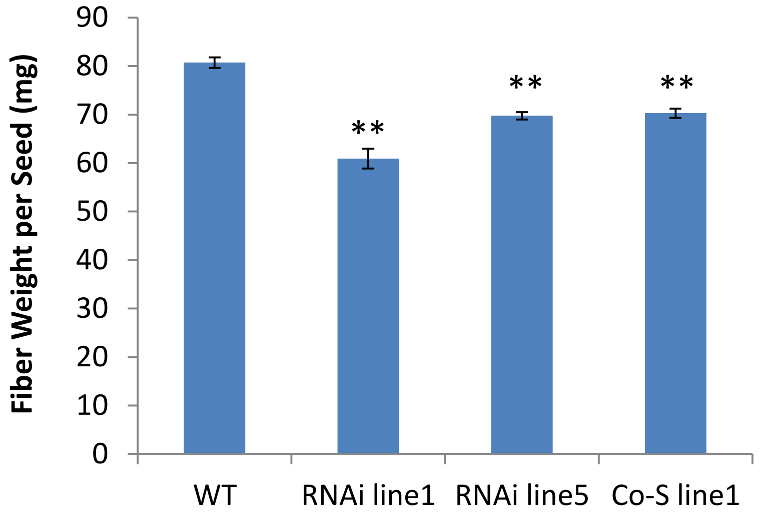

Supplemental Figure S7. Reduced fiber weight in *GhKOR1* down-regulated transgenic cotton. Mature fiber weight per seed of transgenic cotton is significantly reduced as compared to that in the wide type.

** indicate a significant difference to wild type in *t*-test with *P*-value of 0.01.

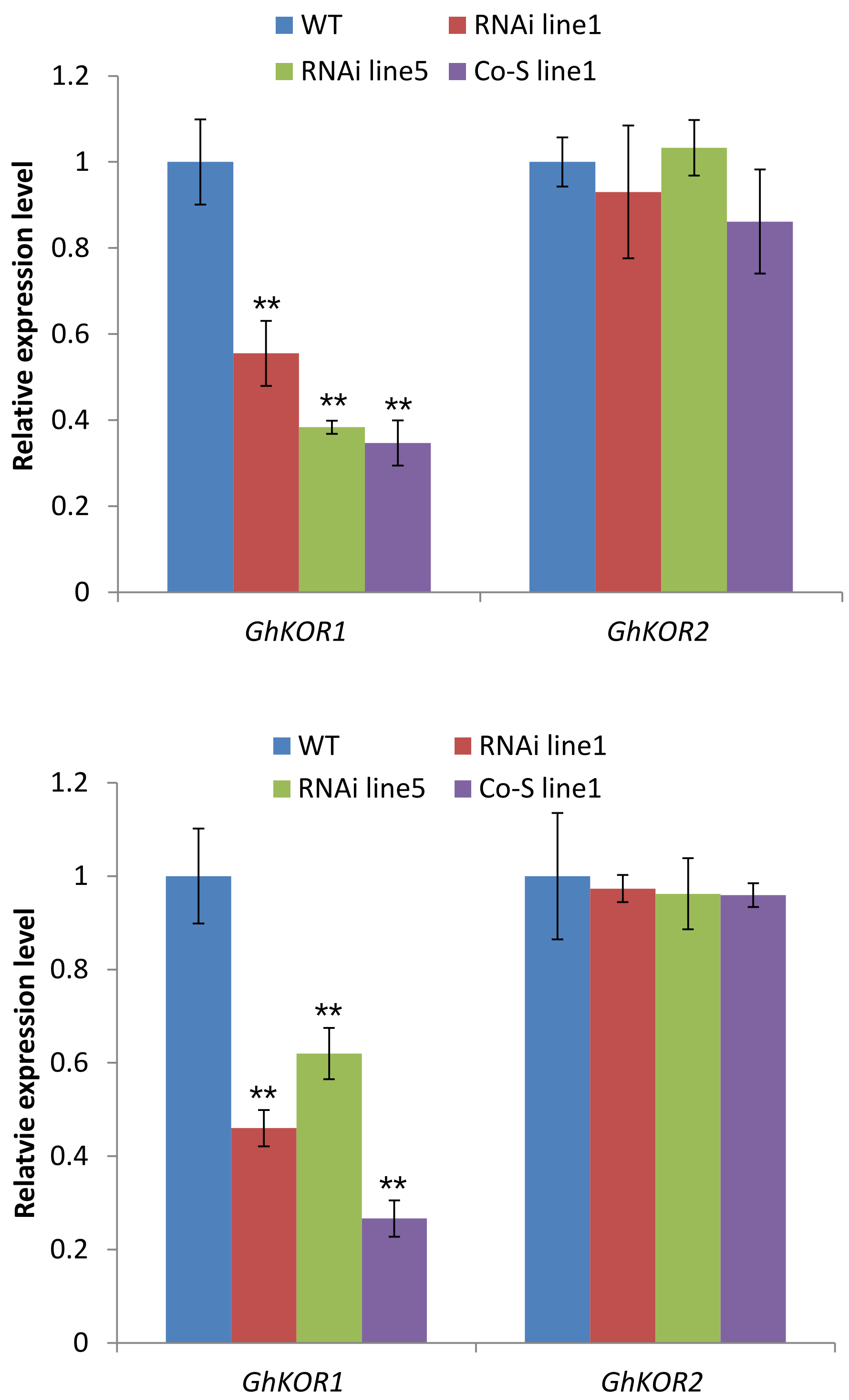

Supplemental Figure S8. Transcript levels of *GhKOR1* and *GhKOR2* in root and above ground tissue of wide type (WT), RNAi and Co-Suppression (Co-S) transgenic cotton.

(A) and (B) qRT-PCR results showing that *GhKOR1* gene was down-regulated in the root (A) and above ground tissue (B) of the three transgenic lines. While *GhKOR2* mRNA level did not appear to exhibit major changes.

** indicate a significant difference to wild type in *t*-test with *P*-value of 0.01. Samples were collected on 30 days after germination (DAG).
